# Antimicrobial resistance in environment and wastewaters of a swine slaughterhouse – tracking ESKAPE bacteria and ESBL-/carbapenemase-producing *Escherichia coli*

**DOI:** 10.3389/fmicb.2026.1957049

**Published:** 2026-10-28

**Authors:** Chiara Manfreda, Flavia Guarneri, Gianluigi Ferri, Chiara Di Vittori, Alberto Olivastri, Maria Antonietta Piccioni, Adriana Ianieri, Giovanni Loris Alborali, Alberto Vergara

**Affiliations:** 1 Department of Food and Drug Sciences, University of Parma, Parma, Italy; 2 “B. Umbertini” Experimental Zooprophylactic Institute of Lombardy and Emilia-Romagna, Brescia, Italy; 3 Department of Veterinary Medicine, “G. Tiecco” Postgraduate Specialization School of Inspection of Food of Animal Origin, University of Teramo, Teramo, Italy; 4 AST Marche, Area Vasta 5, I.A.O.A. Veterinary Service, Ascoli Piceno, Italy

**Keywords:** antimicrobial resistance, environmental contamination, *Escherichia coli*, ESKAPE, one health, slaughterhouse, wastewater, whole-genome sequencing

## Abstract

**Introduction:**

This study investigated the occurrence, antimicrobial resistance (AMR) profiles, and genomic features of selected ESKAPE bacteria and ESBL-/carbapenemase-producing *Escherichia coli* isolated from wastewater and floor swabs in a swine slaughterhouse in Central Italy.

**Methods:**

By integrating microbiological and genomic analyses across different operational phases, the study observed persistence and dissemination of antimicrobial resistant bacteria (ARB) within the abattoir environment.

**Results:**

*E. coli* was the most prevalent species (39.6%) across all matrices, confirming its role as an indicator of fecal and environmental contamination. Despite cleaning and depuration, few ARB and antimicrobial resistance genes (ARGs) persisted, particularly in wastewater, reflecting the partial efficacy of conventional sanitation and treatment measures. The antimicrobial susceptibility tests revealed high resistance against ampicillin (47.2%), tetracycline (41.2%), and trimethoprim (34.0%), with multidrug resistant isolates occurring in nearly one-third of samples. Whole-genome sequencing identified the resistome, e.g., *bla*_TEM_, *bla*_CTX-M_, and *bla*_OXA-48_ genes. The detection of *bla*_NDM_ in treated wastewater underscores the potential for environmental dissemination of carbapenemase producers. Phylogenetic analyses revealed clonal persistence of OXA- and ESBL-producing *E. coli* and *Acinetobacter baumannii* across samples, while livestock-associated MRSA ST398 indicated localized contamination events.

**Discussion:**

These findings demonstrate that slaughterhouse ecosystems can function as active reservoirs and transmission nodes for AMR.

## 1 Introduction

Antimicrobial resistance (AMR) is considered a major global concern (Murray et al., 2022). It phenomenon develops and spreads among the microbiomes of humans, animals, and environment, presenting a multifaceted problem that underscores the relevance of the One Health framework (McEwen and Collignon, 2018). AMR transmission occurs through vertical and horizontal gene transfer (HGT) events mediated by mobile genetic elements (MGEs) such as plasmids, integrons, and transposons. These processes promote the spread of antimicrobial resistance genes (ARGs), contributing to the emergence of multidrug resistant (MDR) bacteria (Partridge et al., 2018). It is widely recognized that the primary driver of this phenomenon is the excessive, inappropriate, or uncontrolled use of antimicrobials in medical and veterinary settings (Zhao et al., 2020).

Research along the swine supply chain, from farms to slaughterhouses, final products and effluents, has shown that each stage of the production process can act as a reservoir for MGEs (Mencía-Ares et al., 2020; Ramesh et al., 2021). Among the most concerning MDR pathogens are the so-called ESKAPE bacteria (*Enterococcus* spp., *Staphylococcus aureus*, *Klebsiella pneumoniae*, *Acinetobacter baumannii*, *Pseudomonas aeruginosa*, *Enterobacter* spp.), as well as ESBL- or carbapenemase-producing *E. coli* (Facciolà et al., 2019; De Oliveira et al., 2020).

In slaughterhouse environments, resistant microorganisms can spread through multiple routes, including the introduction of colonized animals, the use of contaminated water during evisceration, and the contact of carcasses with contaminated surfaces (Savin et al., 2020). Even after wastewater treatment, whether in on-site plants or municipal wastewater treatment plants (mWWTPs), effluents often still contain antimicrobial residues and resistant bacteria (ARBs), contributing to their dissemination in the environment (Müller et al., 2018; Kaesbohrer et al., 2019; Huang et al., 2022). Several studies have documented the presence of ARBs in slaughterhouse effluents, particularly from pig and poultry slaughter facilities. For example, Homeier-Bachmann et al. (2021) discovered ESBL-producing *E. coli* and *K. pneumoniae* and colistin-resistant bacteria in wastewater from pig slaughterhouses, with a high proportion of MDR isolates. In German pig slaughterhouses, ARBs belonging to the ESKAPE group and ESBL-producing *E. coli* have been isolated from multiple stages of processing waters and associated with wastewater, along with detectable antimicrobial residues (Cong et al., 2023). A systematic review, published by Foyle et al. (2023), further highlighted the global distribution of AMR in animal slaughterhouse effluents, with resistance observed against major antimicrobial classes such as aminoglycosides, fluoroquinolones, and β-lactams. Collectively, these findings indicate that slaughterhouse wastewater represents a critical point, and the antibiotic usage in livestock production can lead to environmental dissemination of resistance profiles.

Similarly, surfaces can come into contact with carcasses throughout the slaughter lines contributing to the persistence and spread of ARBs. Furthermore, biofilm formation enhances bacterial survival on stainless steel, plastic, and floor surfaces, protecting cells from disinfectants and promoting gene exchange (Nakanishi et al., 2021; Kaleva et al., 2023). Floors and drainage systems, in particular, act as hotspots where water, blood, and organic residues accumulate, creating niches for ARBs persistence. Studies in swine abattoirs have frequently detected *E. coli*, *Enterococcus* spp., and *S. aureus* from surface and floor swabs, often exhibiting MDR phenotypes, including ESBL-producing *E. coli* and methicillin-resistant *S. aureus* (MRSA) (Ghidini et al., 2022). Recently, Heljanko et al. (2024) conducted a pilot study in South African pig and cattle abattoirs, integrating wastewater and environmental sampling to evaluate AMR dissemination along the slaughter line. The authors detected ESBL-producing *E. coli* and *K. pneumoniae*, along with MRSA, in both wastewater and floor drains, confirming that abattoir environments can act as persistent reservoirs and transmission points for resistant bacteria even after cleaning. This integrated sampling strategy provides a valuable framework for understanding AMR dynamics at the slaughterhouse level and underscores the need for surveillance systems that include both effluent and environmental matrices (Heljanko et al., 2024).

Despite the growing body of international evidence, data on the occurrence and genomic characterization of clinically relevant ARBs, at swine slaughterhouse wastewater level, has been poorly studied in Italy. Indeed, no previous Italian study has comprehensively investigated the occurrence, antimicrobial resistance profiles, and genomic characteristics of ESKAPE bacteria together with ESBL- and carbapenemase-producing *E. coli* in wastewater before and after on-site treatment, while simultaneously including environmental surface sampling within the same slaughterhouse.

Based on this background, this study aims to report the prevalence of selected ESKAPE bacteria, specifically methicillin-resistant *S. aureus* (MRSA), *A. baumannii*, ESBL-/carbapenemase-producing *K. pneumoniae* and *E. coli*, in wastewater and environmental swabs matrices collected in a swine slaughterhouse. Additionally, we evaluated the potential differences in bacterial occurrence before and after disinfection and water depuration processes.

## 2 Materials and methods

### 2.1 Samples collection

Sampling activities were carried out at a swine slaughterhouse located in Central Italy over a period of 6 months (between January and July 2025). Sampling was conducted every 2 weeks, on each occasion both wastewater (WW) and floor swabs (FS) specimens were collected, for a total of 12 sampling days. The wastewater purification plant employed a total oxidation activated sludge biological system before release into the public sewerage, in compliance with the Italian Law No. 152/2006 (2006) which transposes the European directive no. 271/1991. More in detail, this system combines the metabolic actions of aerobic bacterial strains in aerated tanks with denitrification processes through circular and repeated exposures until the final chlorination step (EU Directive 271/1991, 1991).

A total of four wastewater (WW) samples were obtained per sampling day, corresponding to one litre collected before the depuration process (two bottles of 0.5 litres each) and one litre collected after the depuration process (two bottles of 0.5 litres each). In parallel, eight floor swabs (FS) samples were taken from the slaughterhouse floor, specifically two from the pre-sectioning (PRE-SEC) area and two from the post-sectioning (POST-SEC) area, both before the beginning of operations (pre-operations) and after their conclusion (post-operations) (Figure 1). To ensure analytical consistency and reduce variability, the two swabs collected within the same area on the same day were considered as a composite sample and assigned the same identification code. One of the swabs was subjected to microbiological analysis for the detection of *Enterobacteriaceae*, while the other was used for the detection of *S. aureus*, thus yielding 4 FS samples per sampling day.

**Figure 1:**
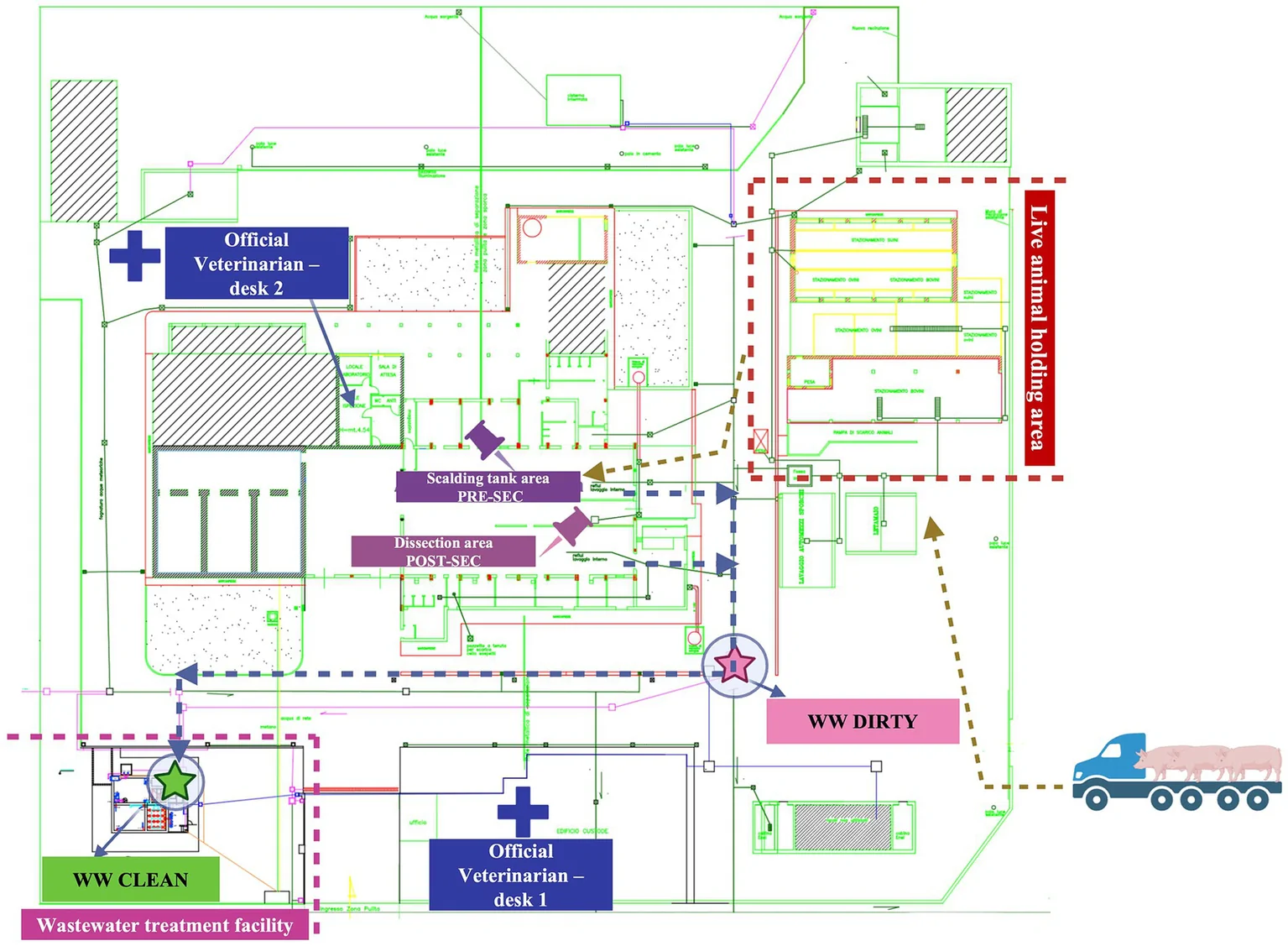
Schematic representation of the sampling protocol at the swine slaughterhouse. The facility is divided into distinct operational areas, including the live animal holding area (outlined by a red dashed line), the scalding tank area PRE-SEC, and the dissection area POST-SEC (marked by purple and dark pink pins, respectively), as well as the wastewater treatment facility (pink dashed line). Wastewater flow is indicated by a blue dashed line. Wastewater (WW) samples were collected before treatment (pink star) and after treatment (green star). Floor swabs (FS) were obtained from the scalding tank area PRE-SEC (purple pin) and the dissection area POST-SEC (dark pink pin).

Therefore, ninety-six (*n* = 96) samples were collected across the 12 sampling days, comprising 48 WW samples (24 before depuration and 24 after depuration processes) and 48 FS samples (24 before operations (PRE-SEC), and 24 after operations (POST-SEC), among the slaughter lines. For the purposes of data interpretation, samples collected before wastewater depuration and after slaughterhouse daily operations were classified as “DIRTY,” whereas those collected after wastewater depuration and before the start of daily operations were classified as “CLEAN” (Table 1).

**Table 1:** Total number of samples per type and category, collected within the considered slaughterhouse.

Sample type	Sampling point	N. of samples	Total over 12 days	CLEAN samples	DIRTY samples	TOTAL/Category
Wastewater	Before depuration	1 ➔ 0.5 litre	12	-	24	48
	WW DIRTY	1 ➔ 0.5 litre	12			
	After depuration	1 ➔ 0.5 litre	12	24	-	
	WW CLEAN	1 ➔ 0.5 litre	12			
Floor swabs	Pre-operations/pre-sectioning	2 swabs ➔ considered as 1	12	24	-	
	FS PRE-SEC CLEAN					
	Pre-operations/post-sectioning	2 swabs ➔ considered as 1	12			48
	FS POST-SEC CLEAN					
	Post-operations/pre-sectioning	2 swabs ➔ considered as 1	12	-	24	
	FS PRE-SEC DIRTY					
	Post-operations/post-sectioning	2 swabs ➔ considered as 1	12			
	FS POST-SEC DIRTY					
TOTAL		8 samples/day	96	48	48	96

WW: wastewater, FS: floor swabs, PRE-SEC: pre-sectioning area, POST-SEC: post-sectioning area, DIRTY: before wastewater depuration and after slaughterhouse daily operations, and CLEAN: samples collected after wastewater depuration and before the start of daily operations.

Immediately following collection, all samples were transferred into sterile sampling bags, stored in refrigerated containers with ice packs, and transported under controlled temperature conditions to the Istituto Zooprofilattico Sperimentale della Lombardia e dell’Emilia Romagna – Brescia headquarters (IZSLER). Microbiological analyses were performed within 24 h from collection to ensure sample integrity and the reliability of the results.

### 2.2 Sample preparation of wastewater

All biological matrices were processed using a standardized diagnostic protocol, which included a pre-enrichment step followed by plating on selective media. For each wastewater sample, 100 mL was filtered using 0.45 μm pore-size cellulose nitrate membrane filter. Although the 0.45 μm filtration can be standardly used for recovering *Enterobacteriaceae* and other Gram-negative strains from water samples, it was demonstrated that any small bacterial strains can potentially pass through the pores ranging between 0.45–0.1 μm (Wang et al., 2007). The filter was then transferred into a stomacher bag containing 9 mL of Buffered Peptone Water (BPW), achieving a 1:10 dilution ratio, and incubated overnight at 37 °C, for the subsequent detection of *A. baumannii*, ESBL-/carbapenemase-producing *E. coli*, indicator *E. coli* and *K. pneumoniae*.

The detection of MRSA in wastewater was not included, as this matrix is highly complex and does not provide information useful for specific risk assessment or for defining targeted control measures. Therefore, surveillance activities were focused on environmental swabs, which allow a more sensitive and meaningful identification of potential surface contaminations and transmission routes related to the environment under investigation.

### 2.3 Sample preparation of environmental swabs, isolation of indicator organisms and Total bacterial count (TBC)

Floor sponge swabs were initially transferred into stomacher bags and weighed before the arrival at the laboratory level to measuring and considering the total mass of soil and debris coming from the floor surfaces; it meant to provide uniform mass samples prior to the pre-enrichments. The MacConkey agar plates were used as non-specific differential medium to perform an initial a preliminary differentiation, of Gram-negative lactose-fermenters, among the *Enterobacteriaceae* (*E. coli* and *K. pneumoniae*). This step was subsequentially followed by the species identification using molecular methods and/or MALDI-TOF MS. The total bacterial count (TBC – expressed as CFU/mL) was performed using the plate count agar which included the incubation at 30.0 °C for 72 h, in agreement with the ISO (International Organization for Standardization) (2013) to determine the total mesophilic count. Bacterial concentrations were calculated as CFU/mL using the standard formula: CFU/mL = (number of colonies)/(inoculation volume × dilution factor). Subsequently, a targeted pre-enrichment phase was performed using appropriate selective enrichment broths in a 1:10 sample-to-medium ratio, depending on the microorganism of interest: Mueller-Hinton Broth for *S. aureus*, and BPW for *A. baumannii*, ESBL-/carbapenemase-producing *E. coli* and *K. pneumoniae*.

### 2.4 Identification of the isolates

Samples enriched with BPW broth were first streaked with a 10 μL loop onto MacConkey agar and incubated overnight at 37 °C for the presumptive identification of indicator *E. Coli* and *K. pneumoniae.* All samples were also streaked onto MacConkey agar supplemented with 1 mg/L cefotaxime and incubated overnight at 42 °C for the preusmptive identification of ESBL-producing *E. Coli* and *K. pneumoniae*, onto Chromid™ Carba smart for the presumptive identification of carbapenemase-producing *E. coli* and *K. pneumoniae* (CPEs), onto ColorexTM Acinetobacter for *Acinetobacter* spp., blood agar and mannitol salt agar for the presumptive isolation of *S. aureus* and MRSA, all incubated overnight at 37 °C. For all positive isolates, 1 to 4 colonies were selected based on purity for further antimicrobial resistance profiling. Presumptive colonies of indicator *E. coli* and ESBL-/carbapenemase producing *E. coli*, identified based on staining and morphological characteristics, were confirmed using the Clermont typing protocol, which detects eight phylogroups by means of a quadruplex end-point PCR (Clermont et al., 2013). Meanwhile, presumptive colonies of indicator, ESBL- and carbapenemase-producing *Klebsiella* spp., *Acinetobacter* spp., and *Staphylococcus* spp. were confirmed using the MALDI-TOF mass spectrometry method (Vitek MS plus, Biomerieux). It is necessary to clarify that, while the MALDI-TOF MS remain highly effective for the routine species-level screening, minor resolution limits do exist compared to the 16S rRNA gene sequencing or full genotyping (Dichtl et al., 2023; Siew et al., 2024). Similarly, although the Illumina, short-read sequencing, delivers high-density core-genome typing, hybrid long-read strategies result often better suited for resolving complex plasmid structures and repetitive mobile genetic elements.

### 2.5 Antimicrobial susceptibility test (AST)

The antimicrobial susceptibility test results were interpreted in accordance with the European Committee on Antimicrobial Susceptibility Testing (EUCAST) and the clinical breakpoints were aligned with the European One Health surveillance framework (EFS/ECDC) to harmonize the monitoring of resistance profiles and traits. All indicator *E. coli* isolates were tested for their susceptibility to 15 different antimicrobials by determination of the minimum inhibitory concentration (MIC) using broth microdilution with commercial plates (Thermo Fisher Scientific, Schwerte, Germany) and the antimicrobial panel EUVSEC3. *S. aureus* strains were tested for their susceptibility to 20 antimicrobial compounds using EUST2 panel, while the remaining isolates were tested against 10 antimicrobials with the antimicrobial panel EUVSEC2. MIC values were interpreted using ECOFF values defined by the European Committee on Antimicrobial Susceptibility Testing (EUCAST, accessed on 10 September 2025). The MIC values below and above the ECOFF values were categorized as “susceptible” and “resistant,” respectively.

### 2.6 Molecular characterization of resistances of *Escherichia coli* and MRSA

Once all the *E. coli* strains were confirmed, the analysis of ESBL resistance genes was performed through a PCR panel allowing identification of *bla*_CTX-M_, *bla*_SHV_, *bla*_TEM_, and *bla*_CMY_ (Fang et al., 2008; Rahman et al., 2013). For carbapenemase-producing isolates, a multiplex PCR panel including *bla*_VIM_, *bla*_IMP_, *bla*_KPC_, *bla*_NDM_, *bla*_OXA-48_, and *bla*_GES-5_ genes was used following an existing protocol (Chamizo-López et al., 2025). Finally, the identification of *mcr* genes, that confer resistance to colistin, was performed using established protocols (Rebelo et al., 2018; Borowiak et al., 2020; Wang et al., 2020). For methicillin- and mupirocin-resistant *S. aureus* (MRSA), detection was performed using a quadruplex PCR assay that simultaneously identifies methicillin and mupirocin resistance and discriminates *S. aureus* from coagulase-negative staphylococci (Zhang et al., 2004).

### 2.7 Genome sequencing

Twenty-two strains, selected based on their antimicrobial resistance patterns and MDR profiles, including isolates obtained up to June 2025, underwent whole-genome sequencing (WGS). Genomic DNA was extracted using the NucleoSpin Tissue Macherey-Nagel Kit and quantified with the QuantiFluor ONE dsDNA System (Promega). Libraries were prepared using the Illumina DNA Prep (M) Tagmentation kit according to the manufacturer’s specifications. Sequencing was performed on an Illumina MiniSeq platform with a High Output cartridge (7.5 Gb capacity) generating 2 × 150 bp paired-end reads. PhiX control DNA was spiked in at 6% for quality monitoring. Raw sequencing data quality was assessed using FastQC (v0.11.9). Comprehensive genomic analyses were conducted using the BV-BRC platform for genome assembly and annotation. Additional analyses including resistome profiling, plasmid identification, k-mer analysis, and SNP calling were performed using the Center for Genomic Epidemiology services, developed by the Technical University of Denmark (DTU) (https://www.genomicepidemiology.org/, accessed on 15 September 2025). All analyses were performed at the Istituto Zooprofilattico Sperimentale della Lombardia ed. Emilia Romagna, within the Genomic Department.

### 2.8 Statistical analysis and visualization

Total bacterial counts of FS samples were log₁₀-transformed [log₁₀(CFU/mL)] to achieve normal distribution for parametric analyses. Descriptive statistics were calculated for all variables by sample category and zone. A two-way analysis of variance (ANOVA) was conducted to examine the effects of sample category (CLEAN vs. DIRTY), zone (PRE-SEC vs. POST-SEC), and their interaction on bacterial counts. Assumptions of normality (Shapiro–Wilk test) and homogeneity of variances (Levene’s test) were verified. Effect sizes were reported as partial eta squared (η^2^) with 95% confidence intervals. For antimicrobial resistance analysis, resistance profiles were visualized using heatmaps, stacked bar plots, and percentage plots. Multi-drug resistance (MDR) patterns (resistance to ≥ 3 antimicrobial classes) were analyzed using descriptive statistics and proportion tests. Statistical analyses were conducted using R statistical software (v 4.2.0) with the following packages: tidyverse v.2.0.0 for data manipulation, ggpubr v.0.6.0 for statistical visualization and tests, car v.3.1.2 for assumption checking, effect size v.1.0.1 for effect size calculation, and patchwork v.1.1.3 for multi-panel figure arrangement.

## 3 Results

A total of 96 samples were collected in an Italian slaughterhouse and processed at IZSLER over a six-month period, from January to July 2025. Fifty percent of the total samples were wastewater (WW) and the other 50% were floor swabs (FS). The floor swabs were evenly collected from the pre-sectioning and post-sectioning areas of the facility, thus forming two distinct spatial groups (PRE-SEC and POST-SEC). Both sample types (WW and FS) were collected before and after cleaning and disinfection procedures, resulting in the classification of samples into two temporal categories, CLEAN and DIRTY.

Two-way ANOVA revealed a highly significant effect of sample category on total bacterial counts (*F*(1,42) = 24.87, *p* < 0.001, η^2^ = 0.37). DIRTY samples exhibited substantially higher bacterial loads (mean log₁₀ CFU/mL = 8.13 ± 1.15) compared to CLEAN samples (mean log₁₀ CFU/mL = 6.59 ± 1.71), representing an approximately 350-fold difference in bacterial concentration. No significant effect of sampling zone (*F*(1,42) = 0.20, *p* = 0.654) or interaction between category and zone (*F*(1,42) = 0.138, *p* = 0.712) was observed, indicating that bacterial contamination did not differ between pre- and post-sectioning areas.

Detailed descriptive statistics for each zone are reported in Table 2, and ANOVA results are summarized in Supplementary Table S1 (see also Figure 2).

**Table 2:** Descriptive statistics of bacterial counts (CFU/mL) by sample category and zone.

Categories	Zones	*n*	Mean CFU/ml	Median CFU/ml	Min CFU/ml	Max CFU/ml	Mean log_10_ CFU/ml	Mean SD log_10_ CFU/ml
CLEAN	POST-SEC	12	3.95 × 10^6^	5.1 × 10^4^	200	3.50 × 10^7^	6.59	1.75
CLEAN	PRE-SEC	12	3.84 × 10^6^	3.6 × 10^4^	400	3.00 × 10^7^	6.58	1.66
DIRTY	POST-SEC	12	7.87 × 10^7^	4.15 × 10^6^	8.00 × 10^5^	7.50 × 10^8^	7.89	0.89
DIRTY	PRE-SEC	12	2.37 × 10^8^	1.10 × 10^7^	2.10 × 10^4^	1.59 × 10^9^	8.37	1.41

WW, wastewater; FS, floor swabs; PRE-SEC, pre-sectioning area; POST-SEC, post-sectioning area; DIRTY, before wastewater depuration and after slaughterhouse daily operations, and CLEAN, samples collected after wastewater depuration and before the start of daily operations.

**Figure 2:**
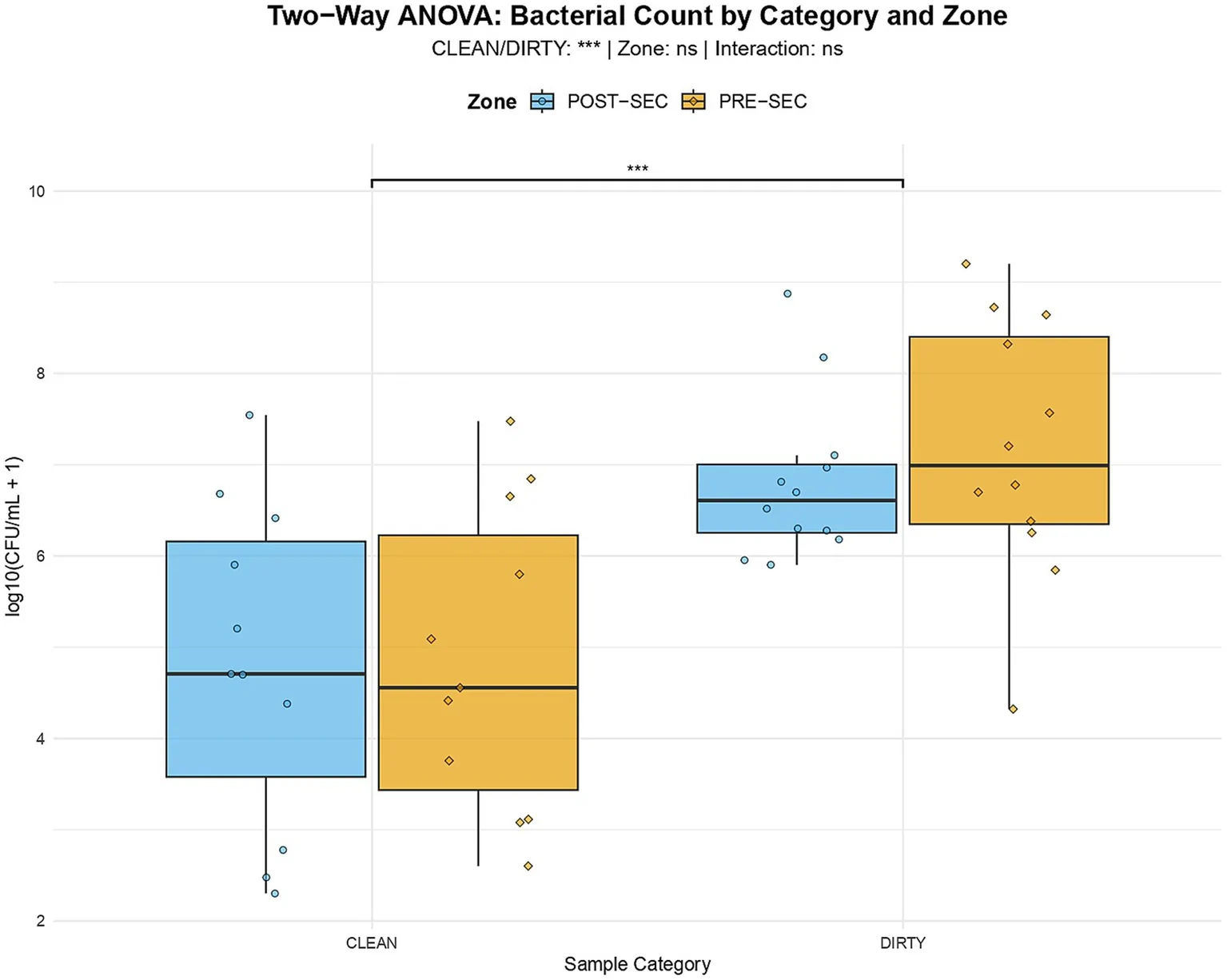
Distribution of bacterial counts across sample categories. Box plots represent log_10_-transformed CFU/mL values. CLEAN samples (mean log_10_ CFU/mL = 4.44) showed significantly lower bacterial loads compared to DIRTY samples (mean log_10_ UFC/mL = 6.99; *p* < 0.001).

### 3.1 Prevalence of positive samples for the investigated bacterial species

A total of 50 samples (52.1%) tested positive for at least one of the investigated microorganisms, yielding 80 isolates overall Table 3. Clear differences were observed both between sample categories (CLEAN vs. DIRTY) and between matrices (WW vs. FS).

**Table 3:** Number of collected samples, positive samples, and the number of the discovered strains.

Samples’ types	Total samples (*n*)	Positive samples (*n*)	Isolates (*n*.)					
			Indicator *E. coli*	*E. coli* ESBL	*E. coli* carbapenemase	*A. baumannii*	Indicator *K. pneumoniae*	*S. aureus* (MRSA)
WW CLEAN	24	14	17	0	2	0	1	–
WW DIRTY	24	20	21	0	6	3	0	–
FS PRE-SEC CLEAN	12	1	0	0	0	0	0	4
FS POST-SEC CLEAN	12	2	1	0	0	0	0	1
FS PRE-SEC DIRTY	12	8	11	1	3	0	0	0
FS POST-SEC DIRTY	12	5	3	2	0	2	0	(2)
*Total*	96	50	53	3	11	5	1	7

WW, wastewater; FS, floor swabs; PRE-SEC, pre-sectioning area; POST-SEC, post-sectioning area; DIRTY, before wastewater depuration and after slaughterhouse daily operations, and CLEAN, samples collected after wastewater depuration and before the start of daily operations.

When considered by matrix, WW samples showed a markedly higher proportion of positives (70.8%, 34/48) compared to FS (33.3%, 16/48).

Within matrices, clear differences were also observed between sample categories: in WW, 83.3% (20/24) of DIRTY samples and 58.3% (14/24) of CLEAN samples were positive, whereas in FS, 54.2% (13/24) of DIRTY and only 12.5% (3/24) of CLEAN samples yielded bacterial growth.

Considering the full dataset (*n* = 48 CLEAN, *n* = 48 DIRTY), indicator *E. coli* was the most frequently detected microorganism, being present in 27.1% of CLEAN samples (*n* = 13/48) and 52.1% of DIRTY samples (*n* = 25/48), corresponding to 39.6% of all tested samples (*n* = 38/96). Indicator *K. pneumoniae* was found only once, in a CLEAN sample (2.1%, *n* = 1/48, 1% overall). Extended-spectrum β-lactamase (ESBL)-producing *E. coli* was rarely identified, with a prevalence of 4.2% in DIRTY samples (*n* = 2/48) and absent in CLEAN; no ESBL-producing *K. pneumoniae* were detected. Carbapenemase-producing *E. coli* was detected in 2% of CLEAN (*n* = 1/48) and 14.6% of DIRTY samples (*n* = 7/48), for an overall prevalence of 8.3% (*n* = 8/96). No carbapenemase-producing *K. pneumoniae* was found. *A. baumannii* was detected exclusively in DIRTY samples (8.3%, *n* = 4/48, 4.1% overall). Finally, *S. aureus* was found in 4.2% of CLEAN samples (*n* = 2/48), while MRSA was identified in 2.1% of DIRTY samples (*n* = 1/48), corresponding to overall prevalences of 2.1 and 1.0%, respectively.

When comparing matrices, indicator *E. coli* prevalence was substantially higher in wastewaters (60.4%, *n* = 29/48) than in floor swabs (18.75%, *n* = 9/48). Carbapenemase-producing *E. coli* were also more frequent in WW (16.7%, *n* = 8/48) compared with FS (2%, *n* = 1/48). In contrast, *S. aureus* isolated from FS was 4.2% (*n* = 2/48), while *Acinetobacter baumannii* was detected in two POST-SEC DIRTY FS samples (16.7%, *n* = 2/12) and in two DIRTY WW samples (8.3%, *n* = 2/24). Within FS, differences emerged between the PRE-SEC and POST-SEC areas. Indicator *E. coli* was more frequently detected in PRE-SEC DIRTY samples (25%, *n* = 6/24) than in POST-SEC DIRTY samples (12.5%, *n* = 3/24). Resistant phenotypes (ESBL-producing *E. coli*, carbapenemase-producing *E. coli*, and MRSA) were detected sporadically in both PRE-SEC and POST-SEC samples, without consistent differences in prevalence.

Despite the relatively limited number of positive samples, multiple isolates were often recovered from individual positive samples. The number of isolates by species and sample type is summarized in Table 3.

### 3.2 Antimicrobial resistance profiles of indicator *E. coli*

All the 53 indicator *E. coli* strains isolated from WW and FS were subjected to antimicrobial susceptibility testing (AST) (Supplementary Table S2). Overall, resistance was most frequently observed to ampicillin (47.2%, 25/53), followed by tetracycline (41.5%, 22/53), trimethoprim (34.0%, 18/53), and chloramphenicol (22.6%, 12/53). Sporadic resistances were detected for tigecycline (5.7%, 3/53), ciprofloxacin (3.8%, 2/53), and azithromycin (3.8%, 2/53). Resistance to last-resort antibiotics, including meropenem and gentamicin, was rare, with only one isolate (1.9%) exhibiting resistance to each (Figure 3).

**Figure 3:**
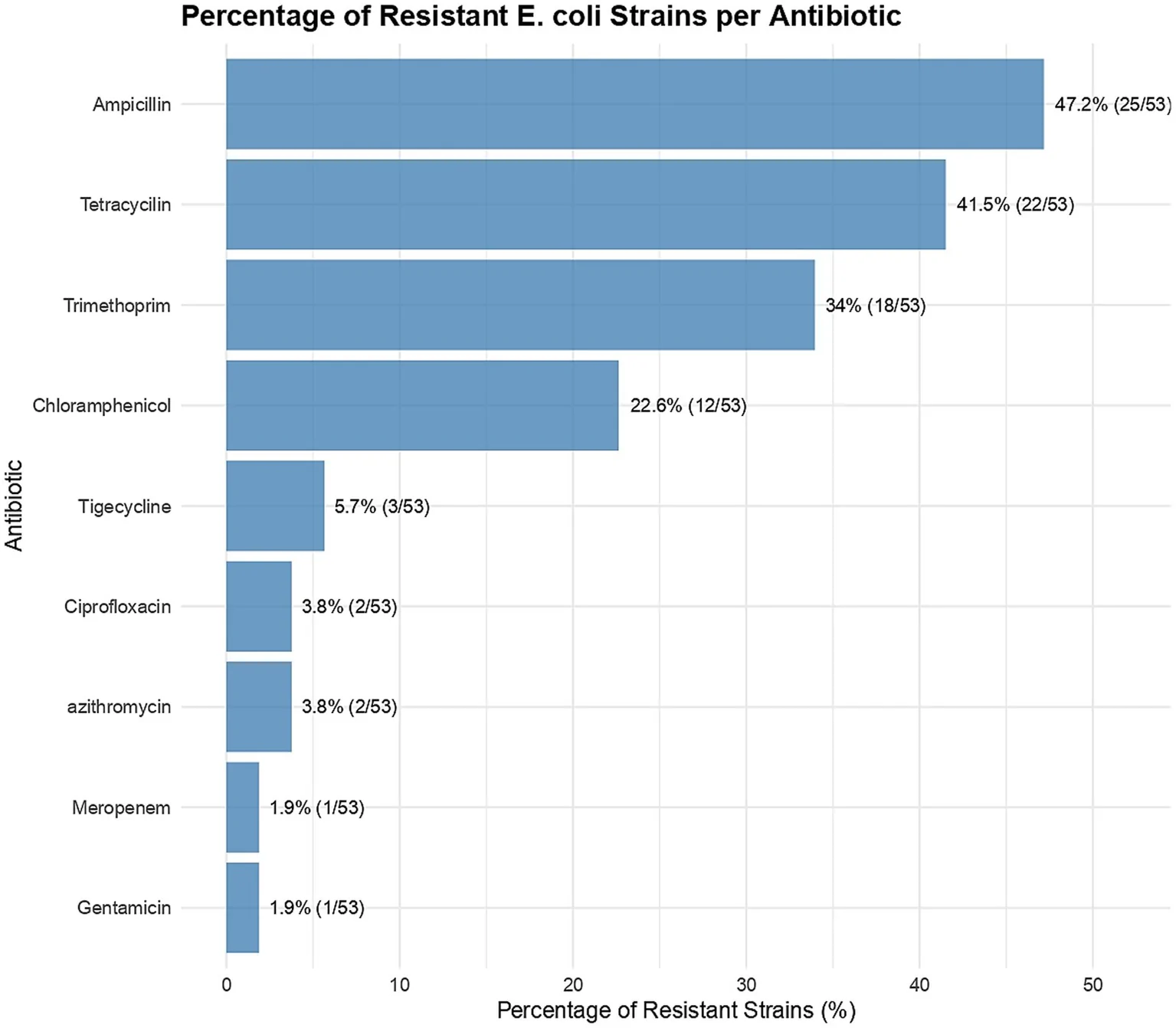
Total resistance to antibiotic molecules among 53 indicator *E. coli*.

Stratification by sample category (CLEAN *n* = 18 vs. DIRTY *n* = 35) revealed distinct resistance profiles. Isolates from DIRTY samples exhibited more frequently resistance to ampicillin (48.6% vs. 44.4%), tetracycline (48.6% vs. 27.8%), trimethoprim (37.1% vs. 27.8%) and chloramphenicol (28.6% vs. 11.1%). An opposite trend was observed for ciprofloxacin and azithromycin, where isolates from CLEAN samples showed a higher resistance rate (5.6%) compared to those from DIRTY samples (2.9%). Tigecycline (8.6%) and meropenem (2.9%) resistance were exclusively observed in DIRTY samples, while gentamicin resistance was found only in CLEAN samples (5.6%). Several antibiotic classes maintained excellent efficacy, with bacterial strains showing complete susceptibility (100%) to amikacin, cefotaxime, ceftazidime, colistin, and nalidixic acid across both environmental categories (Figure 4).

**Figure 4:**
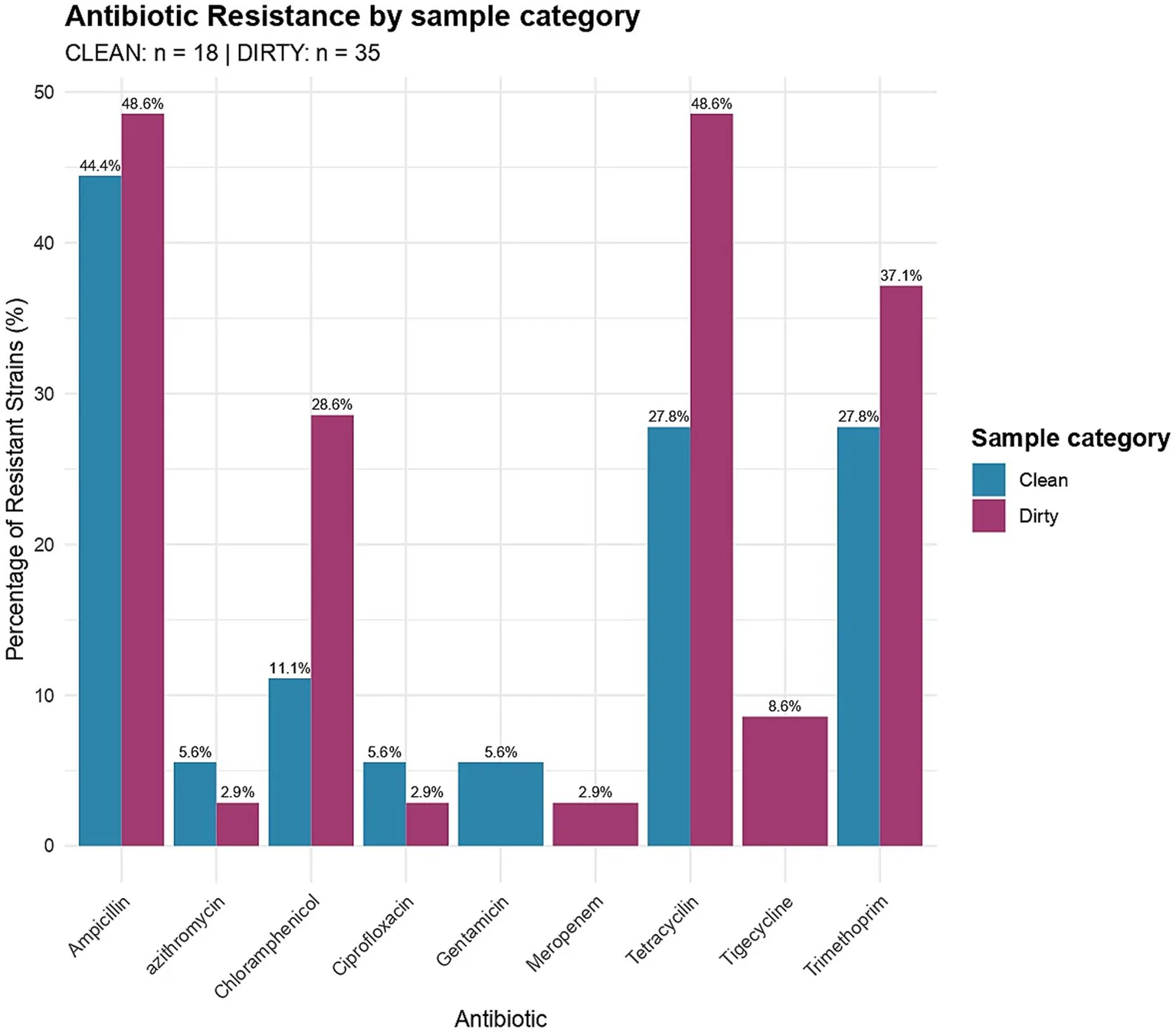
Antibiotic resistance of indicator *E. coli* stratified by sample category (CLEAN vs. DIRTY).

Antimicrobial susceptibility patterns of indicator *E. coli* strains were further examined according to matrix, sampling zone for FS, and category (Figure 5). In WW CLEAN samples, isolates exhibited the highest resistance to ampicillin (47.1%), followed by tetracycline and trimethoprim (29.4% each). Marginal AMR profiles were observed for chloramphenicol (11.8%), ciprofloxacin, gentamicin, and azithromycin (5.9% each). In WW DIRTY samples, resistance was generally higher, with ampicillin (66.7%) and tetracycline (61.9%) showing the highest prevalence, followed by chloramphenicol and trimethoprim (33.3% each). Rare resistance events were observed for ciprofloxacin, tigecycline, meropenem, and azithromycin (4.8% each) (Figure 5A).

**Figure 5:**
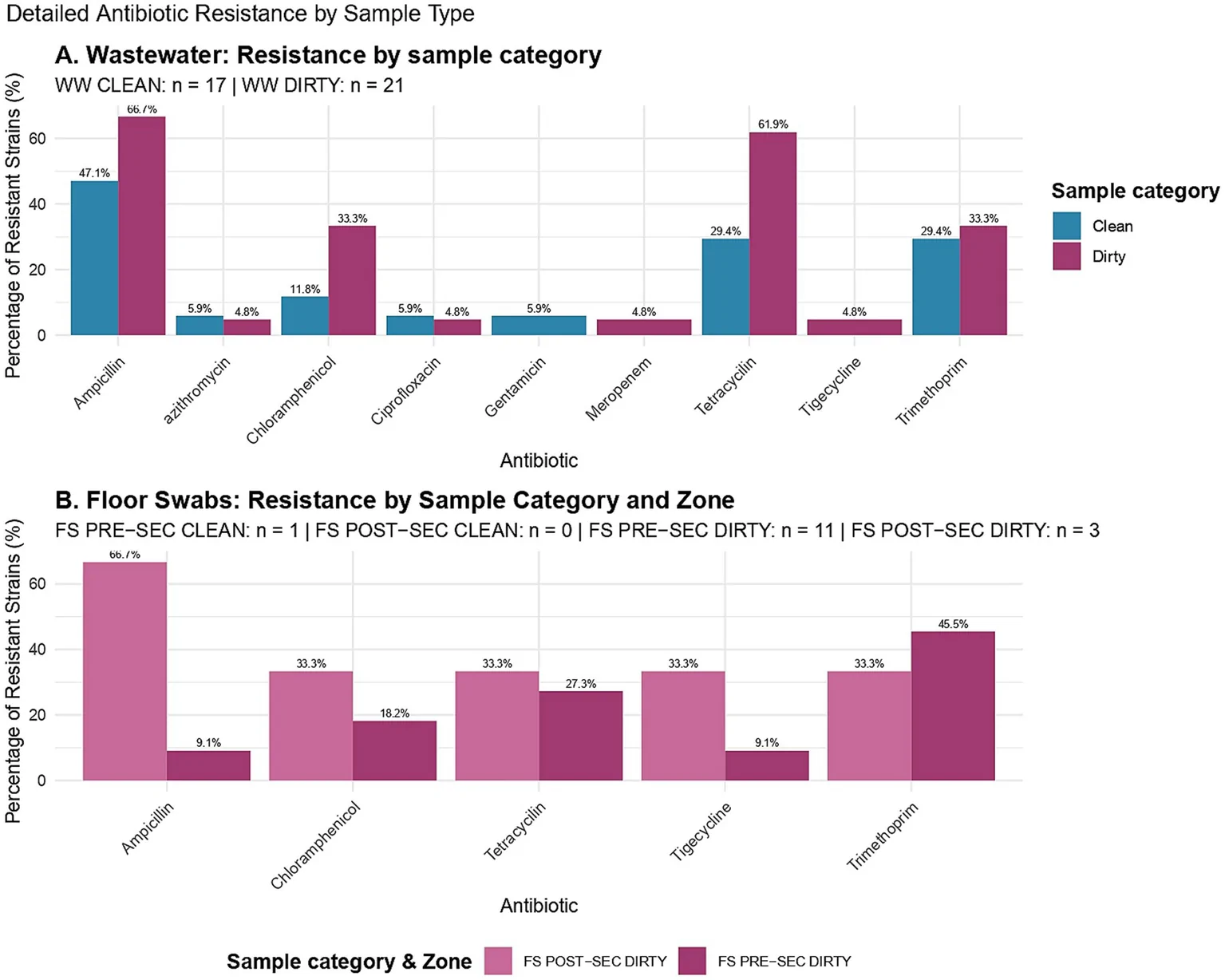
Antibiotic resistance profiles of indicator *E. coli* strains from wastewater and floor swab samples. **(A)** Percentage of resistant strains detected in wastewater samples, divided into clean (WW CLEAN) and dirty (WW DIRTY) categories. **(B)** Percentage of resistant strains isolated from floor swabs divided into before and after sectioning, before and after cleaning categories (FS PRE-SEC CLEAN, FS POST-SEC CLEAN, FS PRE-SEC DIRTY, FS POST-SEC DIRTY). For FS only DIRTY samples exhibited resistances and thus CLEAN FS samples are not provided in the figure.

In FS POST-SEC DIRTY samples, resistance was most frequently detected for ampicillin (66.7%), while resistance to tetracycline, chloramphenicol, trimethoprim, and tigecycline were each observed in 33.3% of isolates each. In FS PRE-SEC DIRTY samples, resistance profiles were more variable, with the highest prevalence recorded for trimethoprim (45.5%), followed by tetracycline (27.3%) and chloramphenicol (18.2%). Ampicillin and tigecycline resistances were less frequent in this group (9.1% each). No resistance was detected in the single *E. coli* strain isolated from FS CLEAN samples (Figure 5B).

Finally, the MDR indicator *E. coli* strains were substantially higher in DIRTY samples (24.5%, *n* = 13/53) than the CLEAN samples (7.5%, *n* = 4/53), resulting in an overall MDR rate of 32% (*n* = 17/53) across all isolates (Figures 6A,B). The proportion of MDR indicator *E. coli* strains was also calculated by sample type, category and zone (Figure 6C). The highest percentage (47.6%) was observed among strains isolated from WW DIRTY, followed by FS POST-SEC DIRTY (33.3%), WW CLEAN (23.5%) and FS PRE-SEC DIRTY (18.2%). Categories in which no MDR strains were detected are not shown in the figure (e.g., FS PRE-SEC CLEAN, FS POST-SEC CLEAN), indicating their absence in floor swabs after cleaning and disinfection procedures, in both plant areas. In detail, ten strains exhibited resistance to three antimicrobial classes, five strains to four classes, and two strains to five classes (Figure 6D).

**Figure 6:**
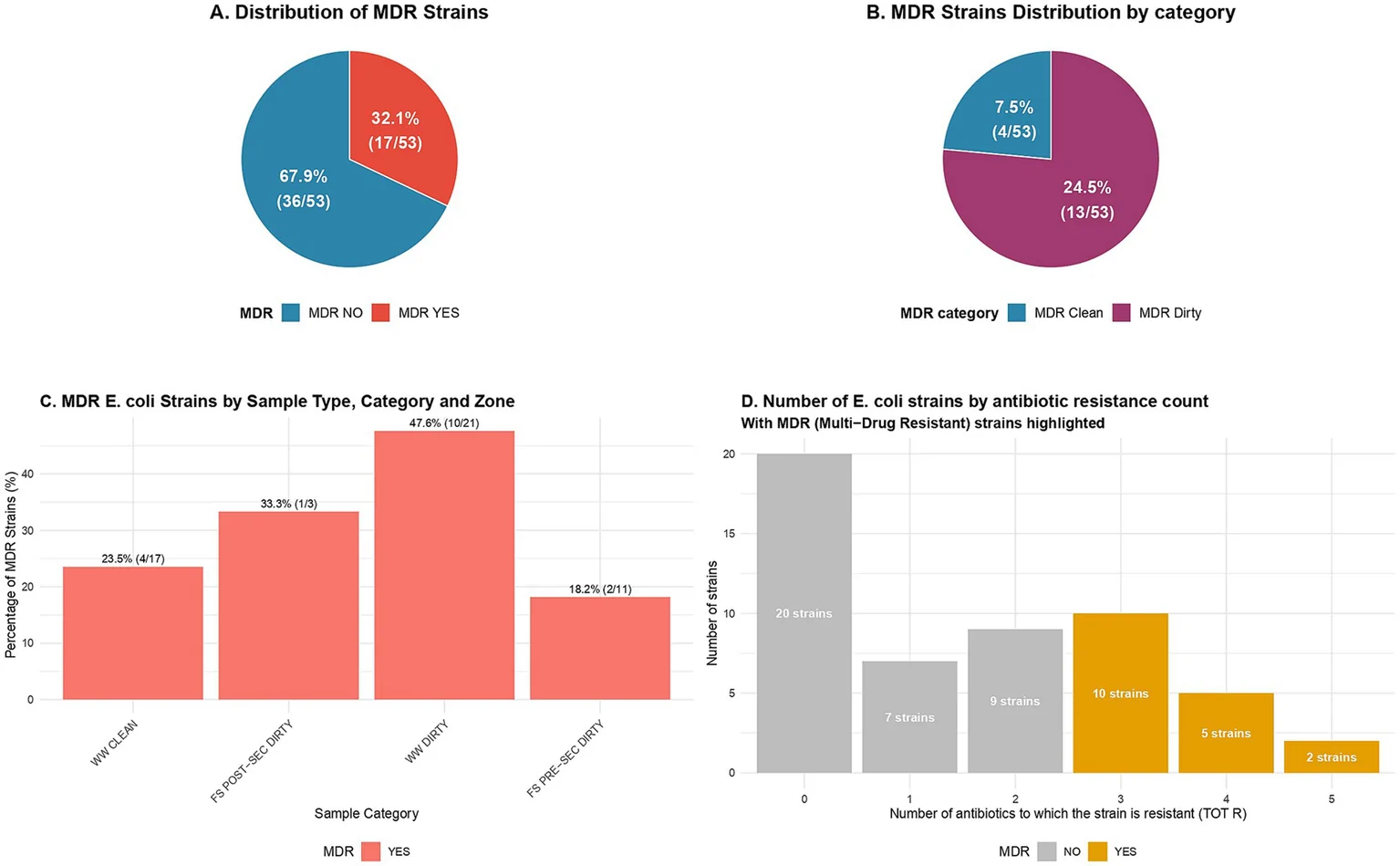
Distribution of MDR indicator *E. coli* isolates among samples. **(A)** Percentage of MDR strains and non-MDR strains. **(B)** Percentage of MDR strains by sample category. **(C)** Percentage of MDR strains by sample type, category, and zone. Only categories where MDR strains were found are shown. **(D)** Number of indicator *E. coli* strains by antibiotic resistance count, with MDR strains highlighted in yellow.

### 3.3 Other isolates characterization

In addition to the 53 indicator *E. coli* strains, the remaining 27 isolates were further characterized for their resistance genes and phenotypic antimicrobial resistance profiles (Figure 7). Overall, the highest prevalence among the isolated strains was observed for carbapenemase-producing *E. coli* strains (13.75%, *n* = 11/80), followed by *S. aureus* (8.75%, *n* = 7/80), *A. baumannii* (6.25%, *n* = 5/80), ESBL-producing *E. coli* (3.75%, *n* = 3/80), and *K. pneumoniae* (1.25%, *n* = 1/80). Among the carbapenemase-producing *E. coli*, ten isolates were recovered from OXA-type selective plates and one from other carbapenemase-producing Enterobacteriaceae selective plate. All OXA-type strains carried the *bla*_OXA-48_ resistance gene, while the other CARBA-type strain harbored the *bla*_NDM_ gene. All ESBL-producing *E. coli* isolates harbored the *bla*_CTX-M_ gene, and one also carried *bla*_TEM_. Among the *S. aureus* isolates, two carried the *mec*A gene and were therefore identified as methicillin-resistant *S. aureus* (MRSA). Phenotypic antimicrobial susceptibility testing revealed variable resistance patterns among the analyzed isolates (Supplementary Tables S3, S4). Among *E. coli* isolates, those carrying *bla*_OXA-48_ were resistant to temocillin and ertapenem, with eight additionally exhibiting resistance to meropenem. The ESBL-producing *E. coli* strains exhibited resistance to cefepime and cefotaxime, and two also showed resistance to ceftazidime. The *E. coli* isolate harboring *bla*_NDM_ displayed the broadest resistance profile, being resistant to ten antimicrobial molecules and four classes, including extended-spectrum cephalosporins, β-lactam/β-lactamase inhibitor combinations, and all tested carbapenems. Conversely, the *K. pneumoniae* isolate was susceptible to all antibiotics tested. Among *A. baumannii* isolates, four out of five were resistant to the ceftazidime/clavulanic acid combination, whereas one strain remained fully susceptible (Supplementary Table S3). Finally, among the seven *S. aureus* isolates analyzed, all exhibited phenotypic resistance to cefoxitin, four isolates (including both MRSA and MSSA) also showed resistance to trimethoprim and quinupristin/dalfopristin, and one strain was resistant to mupirocin. The MRSA isolates displayed an MDR profile, with resistance to cefoxitin, clindamycin, quinupristin/dalfopristin, streptomycin, tetracycline, tiamulin, and trimethoprim; one MRSA isolate also exhibited resistance to erythromycin (Supplementary Table S4).

**Figure 7:**
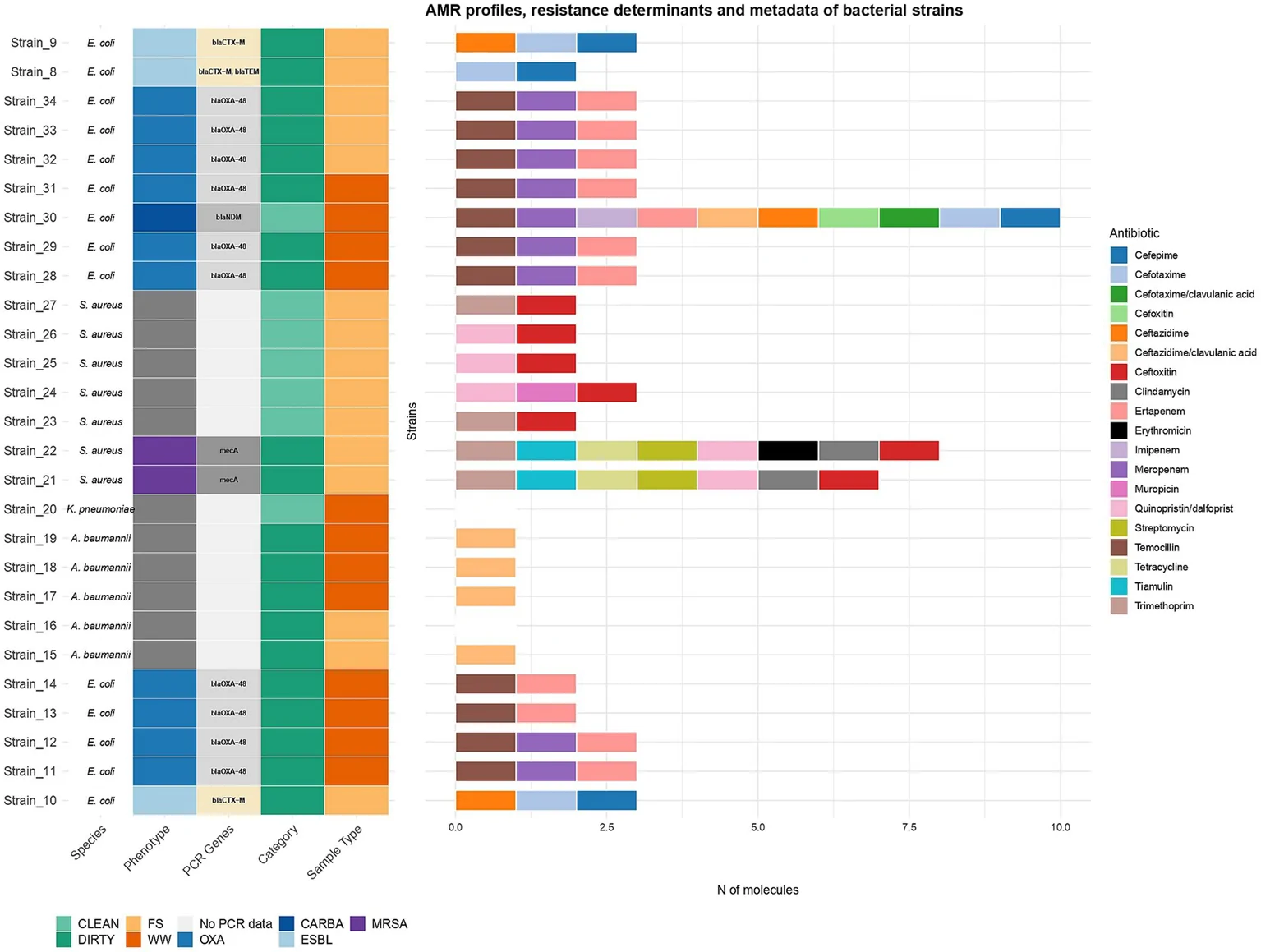
AST results of *S. aureus*, ESBL-/carbapenemase-producing *E. coli*, *K. pneumoniae*, and *A. baumannii*. Additional information about phenotype, PCR genes, sample category, and sample type are added per each strain.

### 3.4 Genomic insights of selected strains

A total of twenty-two bacterial strains were selected for genomic characterization based on their antimicrobial resistance patterns and MDR profiles, including isolates obtained up to June 2025 (Supplementary Table S5). The collection comprised fourteen *E. coli* (seven indicator, three ESBL-producing, and four carbapenemase-producing), five *A. baumannii*, one *K. pneumoniae*, and two MRSA isolates. Whole-genome sequencing (WGS) revealed a diverse array of resistance determinants, showing a strong overall correlation with phenotypic resistance profiles. Aminoglycoside resistance genes (*aad*A1, *aad*A2, *aph*(3′)-Ia, *aph*(3″)-Ib, *aph*(6)-Id, *ant*(3″)-Ia) were widely detected but not phenotypically expressed. Conversely, beta-lactamase genes, including *bla*_TEM-1B_, *bla*_TEM-1C_, *bla*_TEM-176_, *bla*_CTX-M-8_, *bla*_CTX-M-14_, *bla*_OXA-181_, and *bla*_SHV-185_, showed strong concordance with MIC-based resistance to ampicillin, cephalosporins, and carbapenems, particularly among ESBL- and carbapenemase-producing *E. coli* and MRSA isolates. The *A. baumannii* isolates harbored intrinsic beta-lactamase genes (*bla*_OXA-126_, *bla*_OXA-208_, *bla*_OXA-317_, *bla*_ADC-25_), displaying phenotypic resistance to the ceftazidime/clavulanic acid combination; however, one isolate (Strain_16) remained phenotypically susceptible, despite the presence of these genes. The *K. pneumoniae* isolate carried *bla*_SHV-185_, *Oqx*A/B and *fos*A, which are associated to resistance against beta-lactams, quinolones, amphenicol, fosfomycin and folate pathway antagonists, but exhibited no phenotypic resistance. Across all isolates, the presence of tetracycline resistance genes (*tet*(A), *tet*(K), *tet*(M)) and amphenicol resistance genes (*flo*R, *cml*A1) was consistent with MICs for tetracyclines and chloramphenicol, while dfrA and sul genes matched phenotypic resistance to trimethoprim and sulfamethoxazole, across the isolates. Plasmid-mediated quinolone resistance genes (*qnr*S1, *Oqx*A/B) were associated with ciprofloxacin resistance in *E. coli*. In MRSA isolates, *mec*A and *bla*Z confirmed both the genotypic and the phenotypic resistance to beta-lactams, while *erm*(C), *lnu*(B), and *lsa*(E) mediated resistance to lincosamides, pleuromutilins, and streptogramins, all consistent with phenotypic observation.

Plasmid replicon typing identified multiple incompatibility groups among the *E. coli* isolates, including *Inc*F (FIA, FIB, FII), *Inc*X (X1, X3), *Inc*I1-I(α), *Inc*R, and Col-type plasmids (ColpEC648, ColKP3). *Inc*F- and *Inc*X-type plasmids were detected in indicator and ESBL-producing *E. coli* strains, while *Col*KP3 and *Inc*X3 plasmids were found in carbapenemase-producing *E. coli* (Strains_11–14). No plasmid replicons were detected in *A. baumannii* (Strains_15–19) or *K. pneumoniae* (Strain_20). Both *S. aureus* ST398 isolates (Strains_21–22) carried *rep*7a and *rep*US43 plasmid replicons (Supplementary Table S6). Single Nucleotide Polymorphism (SNP)-based phylogenetic analysis of the 14 *E. coli* isolates (Strains_1–14) revealed three distinct clusters corresponding to their resistance phenotypes. The OXA-producing isolates (Strains_11–14) formed a clonal cluster (0–1 SNP differences), comprising three isolates from wastewater (WW) DIRTY samples and one from a WW CLEAN sample (Strain_13), collected across three different sampling days. The ESBL-producing isolates (Strains_9–10) also formed a distinct clonal cluster, separate from the OXA group, and were both recovered from floor swab (FS) DIRTY samples collected on two consecutive months.

The indicator *E. coli* isolates (Strains_1–7) were genetically heterogeneous, showing SNP distances in the thousands to tens of thousands both on themselves and relative to the MDR clusters. An exception was observed for Strain_6, which showed 1,252 allelic differences from the ESBL-producing isolate Strain_8, indicating a degree of genetic relatedness despite being isolated from FS DIRTY samples collected in consecutive months (Supplementary Table S7; Figure 8).

**Figure 8:**
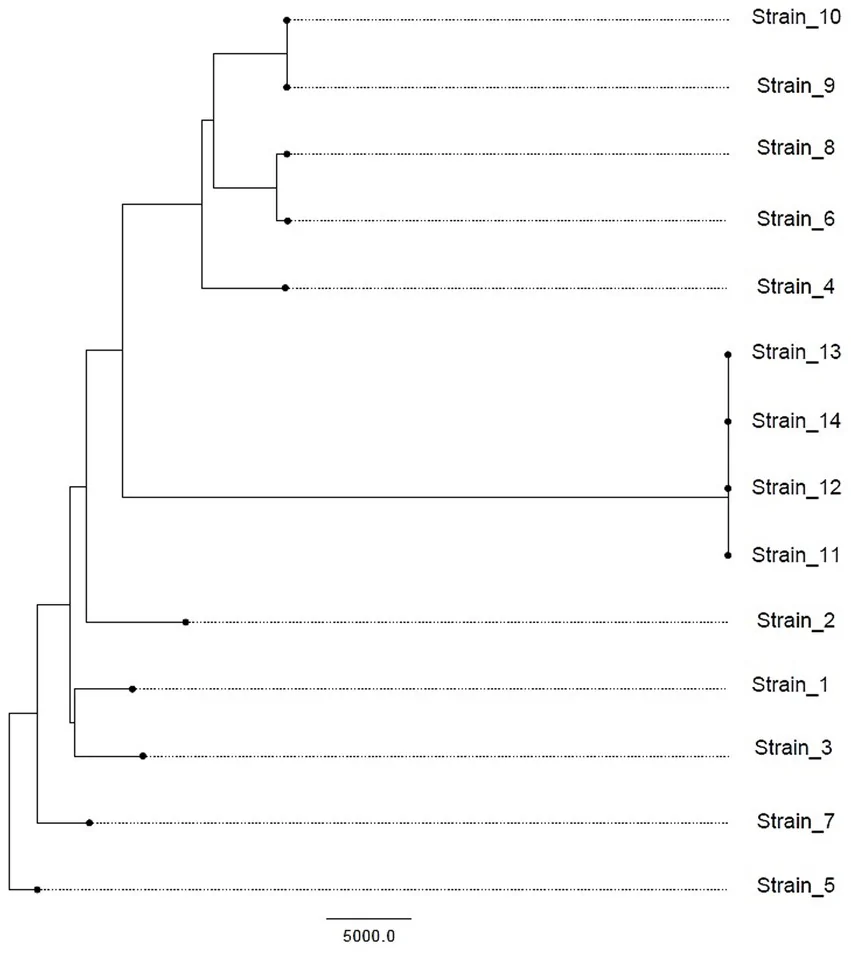
Phylogenetic tree of *E. coli* built with FigTree v1.4.4. Description of strains (Ceppo_n) can be found in Supplementary Table S5.

For *A. baumannii*, Strain_17, Strain_18, and Strain_19, all recovered from two WW DIRTY samples on the same day, were identical at the SNP level (0 SNPs), forming a single clonal cluster. The remaining two isolates were genetically distant from this group and from each other (Supplementary Table S8; Figure 9).

**Figure 9:**
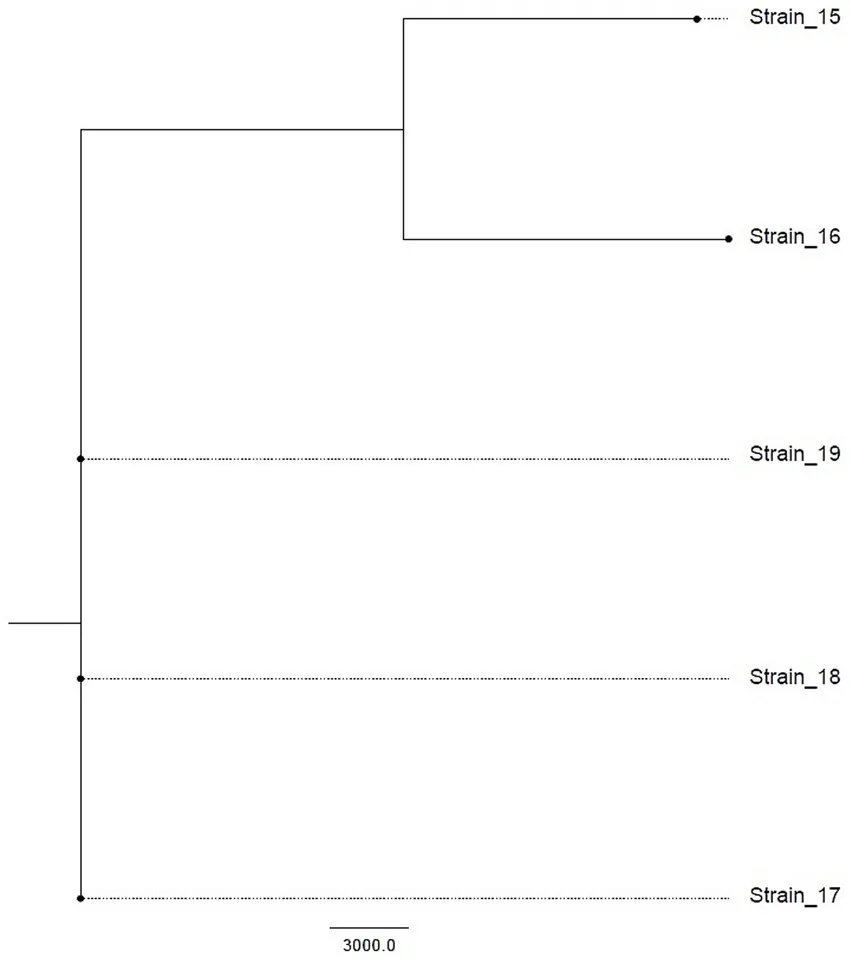
Phylogenetic tree of *A. baumannii* built with FigTree v.1.4.4. Description of strains (Strain_n) can be found in Supplementary Table S5.

The MRSA isolates, which were recovered from the same sample, exhibited only minor genetic differences (21 SNPs), despite slight variations in their phenotypic resistance profiles, thereby confirming their clonal relationship.

## 4 Discussion

This study provides a comprehensive analysis of the occurrence, antimicrobial resistance (AMR) patterns, and genomic features of selected ESKAPE bacteria and ESBL-/carbapenemase-producing *E. coli* isolated from wastewater and environmental surfaces within a swine slaughterhouse located in central Italy. The integrated sampling of both wastewater and floor swabs matrices across multiple operational phases allowed the capture of the dynamics of bacterial persistence and dissemination in a complex processing environment. Overall, the findings confirm that slaughterhouse ecosystems act as significant reservoirs and transmission nodes for antimicrobial resistant bacteria (ARB) and antimicrobial resistance genes (ARGs), even after cleaning and wastewater treatment processes, underscoring the challenges of controlling AMR from a One-Health perspective (McEwen and Collignon, 2018; Heljanko et al., 2024).

To the best of our knowledge, this is the first study conducted in Italy to integrate culture-based microbiology, antimicrobial susceptibility testing, whole-genome sequencing, and phylogenetic analysis to characterize ESKAPE bacteria together with ESBL- and carbapenemase-producing *E. coli* recovered from swine slaughterhouse wastewater before and after on-site wastewater treatment, while also including environmental surfaces sampled before and after sanitation. By combining these complementary approaches, our findings provide novel baseline evidence on the persistence and dissemination of clinically relevant ARBs within the Italian swine slaughterhouse sector.

### 4.1 Environmental prevalence and contamination dynamics

The screened wastewater samples (WW) showed a higher prevalence and harboring frequency (70.8%) of ARBs and ARGs than the environmental floor swab (FS) samples (33.3%). It highlighted the WW samples as critical reservoirs and vector for the AMR diffusion in the slaughterhouse microbiome. Within matrices, the effect of cleaning and disinfection differed markedly: in FS, positivity decreased from 54.2% in DIRTY to 12.5% in CLEAN samples, while in WW the reduction was limited (from 83.3 to 58.3%), indicating persistent microbial contamination despite depuration. These results clearly show that while surface cleaning effectively reduces bacterial loads, wastewater management remains a critical bottleneck, as it supports bacterial survival and the potential recirculation of resistant strains.

The high prevalence of *E. coli* observed in both wastewater and floor swab samples confirms its role as a key indicator species for fecal and environmental contamination within abattoir environments (Kusturov et al., 2017). The marked difference in *E. coli* prevalence between “DIRTY” and “CLEAN” samples, (i.e., before and after sanitation and disinfection procedures), together with the elevated bacterial loads detected through total bacterial count (TBC), reflects both the effectiveness and the limitations, of standard cleaning and depuration processes in such facilities. Wastewater samples, particularly those collected before depuration, represented the most contaminated matrix, consistent with previous reports showing that slaughterhouse effluents often contain high loads of both ARB and residual antimicrobials (Homeier-Bachmann et al., 2021; Foyle et al., 2023). Even after depuration, *E. coli* and carbapenemase-producing *E. coli* were still detected, indicating that bacterial denitrification and chlorination may not fully eliminate resistant strains or ARGs (Savin et al., 2021; Huang et al., 2022). The persistence of *E. coli* and, to a lesser extent *S. aureus* in post-cleaning samples suggests incomplete microbial removal and the possible establishment of biofilm-associated communities in drains and on processing surfaces (Nakanishi et al., 2021; Kaleva et al., 2023). Biofilms are widely recognized as reservoirs of AMR, where microbial cells exhibit from enhanced tolerance to disinfectants and antibiotics and where horizontal gene transfer (HGT) occurs at higher frequencies (Donlan, 2002; Madsen et al., 2012).

### 4.2 Antimicrobial resistance patterns and MDR profiles

Phenotypic antimicrobial susceptibility testing revealed that resistance among indicator *E. coli* isolates was primarily directed toward older and widely used antibiotics, such as ampicillin, tetracycline, and trimethoprim. This trend is consistent with previous surveys of *E. coli* from pig farms and slaughterhouses, where these compounds remain among the most used antimicrobials (Mencía-Ares et al., 2020; Caneschi et al., 2023).

Nevertheless, the detection of MDR isolates in 32% of cases, particularly in wastewater, supports the hypothesis that abattoir effluents serve as hotspots for MDR gene exchange and selection (Savin et al., 2020; Heljanko et al., 2024). While comparative analyses between sample categories revealed higher resistance rates in DIRTY than in CLEAN samples, the persistence of resistance strains and the detection of closely related and/or same clones, among the different sampling sites, could be not exclusively attributed to the environmental persistence of resistant subpopulations. It is also mandatory to consider the different possible cross-contaminations, e.g., vectored by personnel, equipment, and from the drainage of environmental biofilm. In wastewater, the dominance of resistance to β-lactams and tetracyclines reflects historical exposure to these compounds in livestock production (Caneschi et al., 2023; EFSA and ECDC, 2025). Within floor swabs, the presence of resistant *E. coli* in post-sectioning DIRTY samples indicates that cross-contamination from carcasses and organic residues can contribute to the environmental dissemination of ARGs within the facility.

### 4.3 Genotypic–phenotypic correlations and resistome insights

Whole-genome sequencing (WGS) of selected isolates revealed a diverse resistome, with high concordance between phenotypic resistance and the presence of β-lactamase and other ARGs. The detection of *bla*_TEM_, *bla*_CTX-M_, and *bla*_OXA-48_ in *E. coli* isolates confirmed the circulation of extended-spectrum β-lactamases (ESBL) and carbapenemases of clinical relevance, often linked to mobile genetic elements such as *Inc*F and *Inc*X plasmids (Manohar et al., 2020). The presence of *bla*_NDM_ in an after-depuration wastewater sample is particularly concerning, as it suggests potential environmental dissemination of metallo-β-lactamase-producing strains, which have been previously reported mainly in clinical settings (Wu et al., 2019). Interestingly, several aminoglycoside-modifying enzyme genes (*aad*A, *aph*, *ant*) were detected, yet none of the corresponding isolates exhibited phenotypic resistance. Conversely, β-lactamase and tetracycline resistance genes showed strong phenotypic correlation, consistent with high MIC values for these classes. In *A. baumannii*, intrinsic resistance determinants such as *bla*_OXA-126_, *bla*_OXA-208_, and *bla*_ADC-25_ were identified, aligning with the characteristic intrinsic resistance mechanisms of this species (Peleg et al., 2008). The absence of acquired carbapenemases suggests that these isolates belong to environmental rather than clinical lineages. The detection of *S. aureus* ST398 carrying *mec*A, *erm*(C), and *lnu*(B) is noteworthy as this lineage, commonly referred to as livestock-associated MRSA (LA-MRSA), has been frequently associated with pig farming and occupational exposure (Price et al., 2012; Larsen et al., 2017), and its occurrence in the slaughterhouse environment reinforces concerns about zoonotic transmission potential among workers.

The co-occurrence of multiple plasmid incompatibility groups, particularly *Inc*F, *Inc*I, *Inc*X, and Col-type plasmids, indicates a complex plasmid ecology capable of facilitating the exchange of ARGs across bacterial species and strains. *Inc*X3 plasmids, found in carbapenemase-producing *E. coli*, are of epidemiological importance, as they have been implicated in the global dissemination of *bla*_OXA-48-like_ and *bla*_NDM_ carbapenemases (Wu et al., 2019; Guo et al., 2022). Similarly, Col-type plasmids, despite their small size, often function as mobilizable elements that mediate ARGs transfer under environmental conditions (Rozwandowicz et al., 2018).

In *S. aureus*, plasmid replicons *rep*7a and *rep*US43 were detected, consistent with previous studies identifying these plasmids as carriers of resistance genes to macrolides, lincosamides, and pleuromutilins in LA-MRSA ST398 (Argudín et al., 2011; Larsen et al., 2017). Although plasmids were not identified in *A. baumannii* or *K. pneumoniae* isolates, these bacteria still harbored chromosomally encoded resistance determinants, indicating that plasmid-independent mechanisms also contribute to AMR persistence in environmental settings.

To further explore the genetic relatedness, persistence, and possible dissemination pathways of resistant isolates within the slaughterhouse environment, a SNP-based phylogenetic analysis was performed on representative *E. coli*, *A. baumannii*, and *S. aureus* strains.

### 4.4 MLST and phylogenetic analysis

The phylogenetic reconstruction of *E. coli*, *A. baumannii*, and *S. aureus* isolates provided additional insights into the population structure and potential persistence dynamics within the slaughterhouse environment. In *E. coli*, three distinct clusters were identified, corresponding to indicator, ESBL-producing, and OXA-producing phenotypes. The clonal grouping of OXA-producing *E. coli* (Strain_11–14), showing 0–1 SNP differences across samples collected on different days and from both wastewater and floor swabs, strongly suggests the persistence and dissemination of a single resistant clone within the facility. The recovery of one genetically indistinguishable OXA-producing isolate from a CLEAN wastewater sample after sanitation further indicates that this lineage was able to withstand cleaning and depuration procedures, consistent with previous evidence of carbapenemase-producing *E. coli* persistence in industrial wastewater systems (Mairi et al., 2018).

Similarly, the two ESBL-producing *E. coli* isolates (Strains_9–10) formed a separate, homogeneous cluster restricted to DIRTY floor swabs collected over two consecutive months, suggesting localized persistence on processing surfaces rather than reintroduction from external sources. In contrast, the indicator *E. coli* population (Strains_1–7) displayed high genomic diversity, reflecting background environmental variability and likely representing independent introduction events from fecal or environmental sources. The moderate genetic similarity observed between Strain_6 and Strain_8 (1,252 SNPs apart), both from DIRTY floor swabs collected over two consecutive months, may indicate the circulation of related lineages or low-level environmental adaptation rather than direct clonal persistence. It is also mandatory to explicit a crucial aspect that, based on the concept of the high genomic plasticity and mutable accessory genome of the *E. coli*, the persistent inferences can be attributed to the core genome SNP analyses, and it should be interpreted with caution.

The three *A. baumannii* isolates (Strains_17–19) showed 0 SNP differences and were obtained from two distinct DIRTY WW samples collected on the same day. This zero-SNP distance can be considered a confirmation of clonal expansion within a single sampling point and timing moment. The remaining isolates were phylogenetically distant, suggesting multiple, unrelated environmental lineages typical of this species. The MRSA isolates, although exhibiting slight phenotypic differences in resistance profiles, clustered together with only 21 SNPs, indicating clonal origin and likely representing within-sample microevolution, in line with the known genomic stability of livestock-associated *S. aureus* ST398 strains (Price et al., 2012; Larsen et al., 2017).

Overall, the phylogenetic data demonstrate that both *E. coli* and *A. baumannii* can persist and recirculate in the slaughterhouse environment, particularly through wastewater routes, while MRSA appears to be associated with localized contamination events. The coexistence of clonal MDR lineages alongside diverse environmental strains highlights the dual dynamics of persistence and reintroduction that underpin AMR maintenance in slaughterhouse settings.

## 5 Limitation of the study

In this paragraph, were reported the main limitations that should be acknowledged. Concerning the non-sequenced isolates, identification relied primarily on phenotypic characterization and MALDI-TOF MS, which offer slightly lower resolution than full 16S rRNA gene sequencing (Dichtl et al., 2023). In addition, the usage of 0.45 μm pore size for sample filtration might have allowed a small fraction of ultramicrobacteria to pass through (Wang et al., 2007). Regarding genomic analysis, the WGS was focuse on 22 representative multi-drug-resistant isolates using short-read Illumina sequencing. While this approach is highly accurate for the core genome SNP typing, it cannot fully reconstruct complex repetitive plasmid architectures or mobile genetic elements. Given these structural limitations and the high genomic plasticity of *E. coli*, conclusions regarding strain persistence, based on core genome analyses, warrant careful interpretation.

## 6 Conclusion

In conclusion, this study demonstrates that swine slaughterhouse environments and associated wastewater function as reservoirs for antimicrobial resistant bacteria and clinically relevant resistance genes, including ESBL- and carbapenemase-producing *E. coli*, livestock-associated MRSA and *A. baumannii*. Despite standard cleaning, disinfection, and wastewater treatment procedures, both bacterial loads and ARGs persist, highlighting the limitations of current mitigation strategies. Whole-genome sequencing revealed diverse resistance profiles and plasmids, confirming the potential for horizontal gene transfer and the dissemination of multidrug-resistant strains. These findings emphasize the importance of integrated One Health surveillance approach, combining microbiological, molecular, and genomic approaches, to monitor, prevent, and ultimately mitigate the spread of antimicrobial resistance from livestock production to the broader environment.

## Data Availability

The datasets presented in this study can be found in online repositories. The names of the repository/repositories and accession number(s) can be found in the article/Supplementary material.
