# Supplementary material for: Antimicrobial resistance in environment and wastewaters of a swine slaughterhouse – tracking ESKAPE bacteria and ESBL-/carbapenemase-producing Escherichia coli
Source: Front Microbiol. 2026 Oct 28;17:1957049. doi: 10.3389/fmicb.2026.1957049 (PMC13752074; doi:10.3389/fmicb.2026.1957049)
Supplement: Supplementary file 1 [file Supplementary_file_1.docx]

***Supplementary Material***

# 1 Supplementary Tables

**Supplementary Table S 1.** Two-way ANOVA results for the effects of sample category and zone on bacterial counts (log₁₀ UFC/mL).

|  | Df | Sum Sq | Mean Sq | F value | Pr(>F) | Significance |
| --- | --- | --- | --- | --- | --- | --- |
| CLEAN_DIRTY | 1 | 52.97 | 52.97 | 24.867 | 1.11e-05 | *** |
| Zona | 1 | 0.43 | 0.43 | 0.204 | 0.654 |  |
| Category:Zone | 1 | 0.29 | 0.29 | 0.138 | 0.712 |  |
| Residuals | 42 | 89.47 | 3.01 |  |  |  |

Note: Df = degrees of freedom; Sum Sq = sum of squares; Mean Sq = mean square; *** p < 0.001

**Supplementary Table S 2.** Antimicrobial susceptibility results expressed as Minimum Inhibitory Concentration (MIC) values, using EUVSEC 3 panel for broth microdilution AST.

| ID | Species | Matrix | Zone | CLEAN vs DIRTY | Phylogenetic Group | AMK | AMP | AZM | CTX | CAZ | CHL | CIP | COL | GEN | MEM | NAL | SMX | TET | TGC | TMP | TOT R |
| --- | --- | --- | --- | --- | --- | --- | --- | --- | --- | --- | --- | --- | --- | --- | --- | --- | --- | --- | --- | --- | --- |
| Strain_35 | *E. coli* ind | WW |  | DIRTY | B1 | <=4 | >32 | 4 | <=0,25 | <=0,25 | <=8 | <=0,015 | <=1 | <=0,5 | <=0,03 | <=4 | <=8 | >32 | <=0,25 | <=0,25 | 2 |
| Strain_36 | *E. coli* ind | WW |  | DIRTY | A | <=4 | >32 | 4 | <=0,25 | 0,5 | <=8 | <=0,015 | <=1 | <=0,5 | 0,12 | <=4 | <=8 | >32 | <=0,25 | <=0,25 | 3 |
| Strain_1 | *E. coli* ind | WW |  | CLEAN | A | <=4 | >32 | 4 | <=0,25 | <=0,25 | >64 | 0,12 | <=1 | <=0,5 | <=0,03 | <=4 | <=8 | >32 | <=0,25 | >16 | 5 |
| Strain_37 | *E. coli* ind | FS | PRE-SEC | CLEAN | E. CLADES I | <=4 | 4 | 4 | <=0,25 | <=0,25 | <=8 | <=0,015 | <=1 | <=0,5 | <=0,03 | <=4 | <=8 | <=2 | <=0,25 | <=0,25 | 0 |
| Strain_38 | *E. coli* ind | FS | PRE-SEC | DIRTY | A | <=4 | 4 | 4 | <=0,25 | <=0,25 | <=8 | <=0,015 | <=1 | <=0,5 | <=0,03 | <=4 | <=8 | <=2 | <=0,25 | <=0,25 | 0 |
| Strain_2 | *E. coli* ind | FS | POST-SEC | DIRTY | B1 | <=4 | >32 | 8 | <=0,25 | 0,5 | >64 | 0,03 | <=1 | <=0,5 | <=0,03 | <=4 | <=8 | >32 | 0,5 | <=0,25 | 4 |
| Strain_39 | *E. coli* ind | FS | POST-SEC | DIRTY | A | <=4 | 2 | 4 | <=0,25 | <=0,25 | <=8 | <=0,015 | <=1 | <=0,5 | <=0,03 | <=4 | <=8 | <=2 | <=0,25 | 0,5 | 0 |
| Strain_3 | *E. coli* ind | WW |  | DIRTY | A/C | <=4 | >32 | 64 | <=0,25 | <=0,25 | <=8 | <=0,015 | <=1 | <=0,5 | <=0,03 | <=4 | >512 | >32 | <=0,25 | >16 | 4 |
| Strain_40 | *E. coli* ind | WW |  | DIRTY | A | <=4 | <=1 | 8 | <=0,25 | <=0,25 | >64 | <=0,015 | <=1 | <=0,5 | <=0,03 | <=4 | <=8 | <=2 | <=0,25 | 0,5 | 1 |
| Strain_4 | *E. coli* ind | WW |  | DIRTY | A | <=4 | >32 | 8 | <=0,25 | <=0,25 | 16 | <=0,015 | <=1 | <=0,5 | <=0,03 | <=4 | >512 | >32 | <=0,25 | >16 | 4 |
| Strain_41 | *E. coli* ind | WW |  | DIRTY | B1 | <=4 | >32 | 4 | <=0,25 | <=0,25 | <=8 | <=0,015 | <=1 | <=0,5 | <=0,03 | <=4 | <=8 | <=2 | <=0,25 | <=0,25 | 1 |
| Strain_42 | *E. coli* ind | WW |  | CLEAN | B1 | <=4 | >32 | 4 | <=0,25 | <=0,25 | <=8 | <=0,015 | <=1 | <=0,5 | <=0,03 | <=4 | >512 | >32 | <=0,25 | >16 | 3 |
| Strain_43 | *E. coli* ind | WW |  | CLEAN | B1 | <=4 | <=1 | 4 | <=0,25 | <=0,25 | <=8 | <=0,015 | <=1 | <=0,5 | <=0,03 | <=4 | >512 | >32 | <=0,25 | >16 | 2 |
| Strain_44 | *E. coli* ind | WW |  | DIRTY | B1 | <=4 | >32 | <=2 | <=0,25 | <=0,25 | <=8 | <=0,015 | <=1 | <=0,5 | <=0,03 | <=4 | <=8 | <=2 | <=0,25 | <=0,25 | 1 |
| Strain_5 | *E. coli* ind | WW |  | DIRTY | A | <=4 | >32 | 8 | <=0,25 | <=0,25 | >64 | 0,25 | <=1 | <=0,5 | <=0,03 | <=4 | <=8 | >32 | <=0,25 | >16 | 5 |
| Strain_45 | *E. coli* ind | WW |  | DIRTY | B1 | <=4 | >32 | 4 | <=0,25 | <=0,25 | >64 | <=0,015 | <=1 | <=0,5 | <=0,03 | <=4 | <=8 | >32 | <=0,25 | <=0,25 | 3 |
| Strain_46 | *E. coli* ind | WW |  | DIRTY | B1 | <=4 | >32 | 4 | <=0,25 | <=0,25 | >64 | <=0,015 | <=1 | 1 | <=0,03 | <=4 | <=8 | >32 | <=0,25 | <=0,25 | 3 |
| Strain_47 | *E. coli* ind | WW |  | CLEAN | B1 | <=4 | 8 | 8 | <=0,25 | <=0,25 | <=8 | <=0,015 | <=1 | <=0,5 | <=0,03 | <=4 | <=8 | <=2 | <=0,25 | <=0,25 | 1 |
| Strain_48 | *E. coli* ind | WW |  | CLEAN | B1 | <=4 | 2 | 4 | <=0,25 | <=0,25 | <=8 | <=0,015 | <=1 | <=0,5 | <=0,03 | <=4 | <=8 | <=2 | <=0,25 | 0,5 | 0 |
| Strain_49 | *E. coli* ind | WW |  | CLEAN | B1 | <=4 | >32 | 8 | <=0,25 | <=0,25 | <=8 | <=0,015 | <=1 | <=0,5 | <=0,03 | <=4 | >512 | <=2 | <=0,25 | >16 | 2 |
| Strain_50 | *E. coli* ind | WW |  | CLEAN | A/C | <=4 | <=1 | 4 | <=0,25 | <=0,25 | <=8 | <=0,015 | <=1 | <=0,5 | <=0,03 | <=4 | <=8 | <=2 | <=0,25 | <=0,25 | 0 |
| Strain_51 | *E. coli* ind | WW |  | CLEAN | B1 | <=4 | 2 | <=2 | <=0,25 | <=0,25 | <=8 | <=0,015 | <=1 | <=0,5 | <=0,03 | <=4 | <=8 | <=2 | <=0,25 | <=0,25 | 0 |
| Strain_52 | *E. coli* ind | WW |  | CLEAN | B1 | <=4 | 4 | 8 | <=0,25 | <=0,25 | <=8 | <=0,015 | <=1 | <=0,5 | <=0,03 | <=4 | <=8 | <=2 | <=0,25 | <=0,25 | 0 |
| Strain_53 | *E. coli* ind | FS | PRE-SEC | DIRTY | A | <=4 | 4 | <=2 | <=0,25 | <=0,25 | 16 | <=0,015 | <=1 | <=0,5 | <=0,03 | <=4 | >512 | >32 | <=0,25 | >16 | 3 |
| Strain_54 | *E. coli* ind | FS | PRE-SEC | DIRTY | A/C | <=4 | 2 | 4 | <=0,25 | <=0,25 | 16 | <=0,015 | <=1 | 1 | <=0,03 | <=4 | >512 | <=2 | <=0,25 | >16 | 2 |
| Strain_55 | *E. coli* ind | WW |  | DIRTY | B1 | <=4 | 4 | <=2 | <=0,25 | <=0,25 | <=8 | <=0,015 | <=1 | <=0,5 | <=0,03 | <=4 | <=8 | <=2 | <=0,25 | <=0,25 | 0 |
| Strain_56 | *E. coli* ind | WW |  | DIRTY | B1 | <=4 | >32 | <=2 | <=0,25 | <=0,25 | <=8 | <=0,015 | <=1 | 1 | <=0,03 | <=4 | <=8 | <=2 | <=0,25 | 0,5 | 1 |
| Strain_57 | *E. coli* ind | FS | PRE-SEC | DIRTY | E | <=4 | 4 | 4 | <=0,25 | <=0,25 | <=8 | <=0,015 | <=1 | <=0,5 | <=0,03 | <=4 | <=8 | <=2 | <=0,25 | 0,5 | 0 |
| Strain_6 | *E. coli* ind | FS | PRE-SEC | DIRTY | B1 | <=4 | 16 | 4 | <=0,25 | <=0,25 | <=8 | <=0,015 | <=1 | <=0,5 | <=0,03 | <=4 | >512 | >32 | 0,5 | >16 | 4 |
| Strain_58 | *E. coli* ind | WW |  | DIRTY | A/C | <=4 | 32 | 4 | <=0,25 | <=0,25 | <=8 | <=0,015 | <=1 | <=0,5 | <=0,03 | <=4 | >512 | >32 | <=0,25 | >16 | 3 |
| Strain_59 | *E. coli* ind | WW |  | DIRTY | A/C | <=4 | <=1 | <=2 | <=0,25 | <=0,25 | <=8 | <=0,015 | <=1 | <=0,5 | <=0,03 | <=4 | <=8 | 32 | <=0,25 | >16 | 2 |
| Strain_60 | *E. coli* ind | WW |  | DIRTY | A | <=4 | 2 | <=2 | <=0,25 | <=0,25 | <=8 | <=0,015 | <=1 | <=0,5 | <=0,03 | <=4 | <=8 | <=2 | <=0,25 | <=0,25 | 0 |
| Strain_61 | *E. coli* ind | WW |  | DIRTY | A/C | <=4 | <=1 | <=2 | <=0,25 | <=0,25 | <=8 | <=0,015 | <=1 | <=0,5 | <=0,03 | <=4 | <=8 | >32 | 0,5 | <=0,25 | 2 |
| Strain_62 | *E. coli* ind | WW |  | CLEAN | A/C | <=4 | <=1 | 4 | <=0,25 | <=0,25 | <=8 | <=0,015 | <=1 | <=0,5 | <=0,03 | <=4 | <=8 | <=2 | <=0,25 | <=0,25 | 0 |
| Strain_63 | *E. coli* ind | WW |  | CLEAN | B1 | <=4 | 4 | 4 | <=0,25 | <=0,25 | <=8 | <=0,015 | <=1 | <=0,5 | <=0,03 | <=4 | >512 | <=2 | <=0,25 | >16 | 0 |
| Strain_64 | *E. coli* ind | WW |  | CLEAN | B1 | <=4 | >32 | <=2 | <=0,25 | <=0,25 | <=8 | <=0,015 | <=1 | <=0,5 | <=0,03 | <=4 | <=8 | <=2 | <=0,25 | <=0,25 | 1 |
| Strain_65 | *E. coli* ind | FS | PRE-SEC | DIRTY | A/C | <=4 | 4 | 4 | <=0,25 | <=0,25 | <=8 | <=0,015 | <=1 | <=0,5 | <=0,03 | <=4 | <=8 | <=2 | <=0,25 | 0,5 | 0 |
| Strain_66 | *E. coli* *ind* | FS | PRE-SEC | DIRTY | A/C | <=4 | <=1 | <=2 | <=0,25 | <=0,25 | <=8 | <=0,015 | <=1 | <=0,5 | <=0,03 | <=4 | <=8 | <=2 | <=0,25 | >16 | 1 |
| Strain_67 | *E. coli* *ind* | FS | PRE-SEC | DIRTY | A/C | <=4 | 2 | 4 | <=0,25 | <=0,25 | <=8 | <=0,015 | <=1 | <=0,5 | <=0,03 | <=4 | <=8 | 16 | <=0,25 | >16 | 2 |
| Strain_68 | *E. coli* *ind* | FS | PRE-SEC | DIRTY | B1 | <=4 | 4 | <=2 | <=0,25 | <=0,25 | <=8 | <=0,015 | <=1 | <=0,5 | <=0,03 | <=4 | <=8 | <=2 | <=0,25 | <=0,25 | 0 |
| Strain_69 | *E. coli* *ind* | WW |  | DIRTY | B1 | <=4 | >32 | 4 | <=0,25 | 1 | <=8 | <=0,015 | <=1 | 1 | <=0,03 | <=4 | >512 | >32 | <=0,25 | >16 | 3 |
| Strain_70 | *E. coli* *ind* | WW |  | CLEAN | A | <=4 | >32 | <=2 | <=0,25 | <=0,25 | <=8 | <=0,015 | <=1 | 1 | <=0,03 | <=4 | <=8 | >32 | <=0,25 | 0,5 | 2 |
| Strain_7 | *E. coli* *ind* | WW |  | DIRTY | A | <=4 | >32 | <=2 | <=0,25 | <=0,25 | >64 | <=0,015 | <=1 | <=0,5 | <=0,03 | <=4 | <=8 | >32 | <=0,25 | >16 | 4 |
| Strain_71 | *E. coli* *ind* | WW |  | CLEAN | A/C | <=4 | 2 | 4 | <=0,25 | <=0,25 | <=8 | <=0,015 | <=1 | <=0,5 | <=0,03 | <=4 | <=8 | <=2 | <=0,25 | <=0,25 | 0 |
| Strain_72 | *E. coli* *ind* | WW |  | CLEAN | B2 | <=4 | 2 | 4 | <=0,25 | <=0,25 | <=8 | <=0,015 | <=1 | 1 | <=0,03 | <=4 | <=8 | <=2 | <=0,25 | <=0,25 | 0 |
| Strain_73 | *E. coli* *ind* | FS | PRE-SEC | DIRTY | A/C | <=4 | 4 | <=2 | <=0,25 | <=0,25 | <=8 | <=0,015 | <=1 | <=0,5 | <=0,03 | <=4 | <=8 | <=2 | <=0,25 | 0,5 | 0 |
| Strain_74 | *E. coli* *ind* | FS | POST-SEC | DIRTY | B1 | <=4 | >32 | 8 | <=0,25 | <=0,25 | <=8 | <=0,015 | <=1 | <=0,5 | <=0,03 | <=4 | >512 | <=2 | <=0,25 | >16 | 2 |
| Strain_75 | *E. coli* *ind* | WW |  | DIRTY | E/D | <=4 | <=1 | 4 | <=0,25 | <=0,25 | <=8 | <=0,015 | <=1 | 1 | <=0,03 | <=4 | <=8 | <=2 | <=0,25 | <=0,25 | 0 |
| Strain_76 | *E. coli* *ind* | WW |  | DIRTY | A/C | <=4 | 2 | <=2 | <=0,25 | <=0,25 | <=8 | <=0,015 | <=1 | <=0,5 | <=0,03 | <=4 | <=8 | <=2 | <=0,25 | <=0,25 | 0 |
| Strain_77 | *E. coli* *ind* | WW |  | CLEAN | A | <=4 | >32 | <=2 | <=0,25 | <=0,25 | 32 | <=0,015 | <=1 | 16 | <=0,03 | <=4 | <=8 | <=2 | <=0,25 | 0,5 | 3 |
| Strain_78 | *E. coli* *ind* | FS | PRE-SEC | DIRTY | A | <=4 | 2 | 8 | <=0,25 | <=0,25 | <=8 | 0,03 | <=1 | <=0,5 | <=0,03 | <=4 | <=8 | <=2 | <=0,25 | <=0,25 | 0 |
| Strain_79 | *E. coli* *ind* | WW |  | DIRTY | E. CLADES I | <=4 | >32 | 4 | <=0,25 | <=0,25 | >64 | <=0,015 | <=1 | 2 | <=0,03 | <=4 | <=8 | >32 | <=0,25 | <=0,25 | 3 |
| Strain_80 | *E. coli* *ind* | WW |  | CLEAN | A | <=4 | >32 | 16 | <=0,25 | <=0,25 | <=8 | <=0,015 | <=1 | 1 | <=0,03 | <=4 | <=8 | >32 | <=0,25 | 0,5 | 3 |

Note: Antibiotic abbreviations used in this study are as follows: AMK, amikacin; AMP, ampicillin; AZM, azithromycin; FEP, cefepime; CTX, cefotaxime; CAZ, ceftazidime; CHL, chloramphenicol; CIP, ciprofloxacin; COL, colistin; GEN, gentamicin; MEM, meropenem; NAL, nalidixic acid; SMX, sulfamethoxazole; TET, tetracycline; TGC, tigecycline; TMP, trimethoprim.

**Supplementary Table S3.** Antimicrobial susceptibility results expressed as Minimum Inhibitory Concentration (MIC) values, using EUVSEC 2 panel for broth microdilution AST.

| ID | Species | Matrix | Zone | CLEAN vs DIRTY | FEP | CTX | CTX/CLA | FOX | CAZ | CAZ/CLA | ETP | IMP | MEM | TEMO | TOT R |
| --- | --- | --- | --- | --- | --- | --- | --- | --- | --- | --- | --- | --- | --- | --- | --- |
| Strain_20 | *K. penumoniae* | WW |  | CLEAN | <=0,06 | <=0,25 | <=0,06 | 2 | <=0,25 | <=0,12 | <=0,015 | 0,25 | <=0,03 | 2 | 0 |
| Strain_8 | *E. coli* ESBL | FS | PRE-SEC | DIRTY | 4 | 8 | <=0,06 | 1 | 0,5 | <=0,12 | <=0,015 | 0,25 | <=0,03 | 4 | 2 |
| Strain_9 | *E. coli* ESBL | FS | POST-SEC | DIRTY | 8 | 16 | <=0,06 | 4 | 1 | <=0,12 | <=0,015 | <=0,12 | <=0,03 | 8 | 3 |
| Strain_10 | *E. coli* ESBL | FS | POST-SEC | DIRTY | 2 | 16 | 0,12 | 4 | 1 | 0,25 | <=0,015 | <=0,12 | <=0,03 | 8 | 3 |
| Strain_30 | *E. coli* CARBA | WW |  | CLEAN | >32 | >64 | >64 | >64 | >128 | >128 | >2 | 4 | 16 | >128 | 10 |
| Strain_11 | *E. coli* OXA | WW |  | DIRTY | 0,12 | <=0,25 | 0,25 | 4 | <=0,25 | <=0,12 | 0,5 | 0,25 | 0,12 | 128 | 3 |
| Strain_12 | *E. coli* OXA | WW |  | DIRTY | 0,12 | <=0,25 | 0,25 | 4 | <=0,25 | 0,25 | 0,5 | 0,25 | 0,12 | 128 | 3 |
| Strain_13 | *E. coli* OXA | WW |  | DIRTY | 0,12 | <=0,25 | 0,25 | 4 | <=0,25 | <=0,12 | 0,5 | 0,25 | 0,06 | 128 | 2 |
| Strain_14 | *E. coli* OXA | WW |  | DIRTY | 0,12 | <=0,25 | 0,25 | 4 | <=0,25 | <=0,12 | 1 | 0,5 | 0,06 | >128 | 2 |
| Strain_28 | *E. coli* OXA | WW |  | DIRTY | 0,12 | <=0,25 | 0,25 | 4 | <=0,25 | <=0,12 | 0,5 | 0,25 | 0,12 | 128 | 3 |
| Strain_29 | *E. coli* OXA | WW |  | DIRTY | 0,12 | <=0,25 | 0,25 | 4 | <=0,25 | <=0,12 | 0,25 | 0,25 | 0,25 | 128 | 3 |
| Strain_31 | *E. coli* OXA | WW |  | DIRTY | 0,12 | <=0,25 | 0,25 | 4 | <=0,25 | 0,25 | 1 | 0,5 | 0,25 | >128 | 3 |
| Strain_32 | *E. coli* OXA | FS | PRE-SEC | DIRTY | 0,12 | <=0,25 | 0,25 | 4 | <=0,25 | <=0,12 | 0,5 | 0,5 | 0,25 | >128 | 3 |
| Strain_33 | *E. coli* OXA | FS | PRE-SEC | DIRTY | 0,12 | <=0,25 | 0,25 | 4 | <=0,25 | <=0,12 | 0,5 | 0,25 | 0,25 | >128 | 3 |
| Strain_34 | *E. coli* OXA | FS | PRE-SEC | DIRTY | 0,12 | <=0,25 | 0,25 | 4 | <=0,25 | <=0,12 | 1 | 0,5 | 0,25 | >128 | 3 |
| Strain_15 | *A. baumannii* | FS | POST-SEC | DIRTY | 1 | 16 | 4 | 32 | 4 | 2 | 1 | 0,25 | 0,12 | 128 | 1 |
| Strain_16 | *A. baumannii* | FS | POST-SEC | DIRTY | 0,5 | 2 | 2 | 32 | 1 | 1 | 1 | 0,25 | 0,25 | 64 | 0 |
| Strain_17 | *A. baumannii* | WW |  | DIRTY | 2 | 8 | 4 | 64 | 2 | 2 | 2 | 0,25 | 0,25 | >128 | 1 |
| Strain_18 | *A. baumannii* | WW |  | DIRTY | 1 | 4 | 4 | 64 | 2 | 4 | 2 | 0,25 | 0,25 | >128 | 1 |
| Strain_19 | *A. baumannii* | WW |  | DIRTY | 2 | 8 | 4 | 64 | 2 | 2 | 2 | 0,25 | 0,25 | >128 | 1 |

Note: ESBL, OXA, CARBA represent the phenotypes of E. coli strains, which was established by growth onto selective plates. Antibiotic abbreviations used in this study are as follows: FEP, cefepime; CTX, cefotaxime; CTX/CLA, cefotaxime/clavulanic acid; FOX, cefoxitin; CAZ, ceftazidime; CAZ/CLA, ceftazidime/clavulanic acid; ETP, ertapenem; IMP, imipenem; MEM, meropenem; TEMO, temocillin.

**Supplementary Table S4.** Antimicrobial susceptibility results expressed as Minimum Inhibitory Concentration (MIC) values, using EUST 2 panel for broth microdilution AST.

|  | Species | Matrix | Zone | CLEAN vs  DIRTY | FOX | CHL | CIP | CLI | ERY | FUS | GEN | KAN | LZD | MUP | HLMUP | PEN | Q/D | RIF | STR | SMX | TET | TIA | TMP | VAN | TOT R |
| --- | --- | --- | --- | --- | --- | --- | --- | --- | --- | --- | --- | --- | --- | --- | --- | --- | --- | --- | --- | --- | --- | --- | --- | --- | --- |
| Strain_23 | *S. aureus* | FS | PRE-SEC | CLEAN | 4 | 8 | <=0,25 | <=0,12 | 0,5 | <=0,25 | <=0,5 | <=4 | 2 | <=0,5 | <=256 | <=0,06 | <=0,5 | <=0,015 | <=4 | <=64 | <=0,5 | <=0,5 | 2 | <=1 | 2 |
| Strain_24 | *S. aureus* | FS | PRE-SEC | CLEAN | 4 | 8 | <=0,25 | <=0,12 | 0,5 | <=0,25 | <=0,5 | <=4 | 2 | 1 | <=256 | <=0,06 | 1 | <=0,015 | <=4 | <=64 | <=0,5 | <=0,5 | <=1 | <=1 | 3 |
| Strain_25 | *S. aureus* | FS | PRE-SEC | CLEAN | 4 | 8 | <=0,25 | <=0,12 | <=0,25 | <=0,25 | <=0,5 | <=4 | 2 | <=0,5 | <=256 | <=0,06 | 1 | <=0,015 | <=4 | <=64 | <=0,5 | 1 | <=1 | <=1 | 2 |
| Strain_26 | *S. aureus* | FS | PRE-SEC | CLEAN | 4 | 8 | <=0,25 | <=0,12 | 0,5 | <=0,25 | <=0,5 | <=4 | 2 | <=0,5 | <=256 | 0,25 | 1 | <=0,015 | <=4 | <=64 | <=0,5 | 1 | <=1 | <=1 | 2 |
| Strain_21 | MRSA | FS | POST-SEC | DIRTY | 16 | 8 | <=0,25 | 1 | 0,5 | <=0,25 | <=0,5 | <=4 | 2 | <=0,5 | <=256 | >1 | 2 | <=0,015 | 32 | <=64 | >16 | 4 | >16 | <=1 | 7 |
| Strain_22 | MRSA | FS | POST-SEC | DIRTY | 8 | 8 | <=0,25 | 4 | >8 | <=0,25 | <=0,5 | <=4 | <=1 | <=0,5 | <=256 | >1 | >4 | <=0,015 | >32 | <=64 | >16 | >4 | >16 | <=1 | 8 |
| Strain_27 | *S. aureus* | FS | POST-SEC | CLEAN | 4 | 8 | <=0,25 | <=0,12 | 0,5 | <=0,25 | <=0,5 | <=4 | 2 | <=0,5 | <=256 | <=0,06 | <=0,5 | <=0,015 | <=4 | <=64 | <=0,5 | <=0,5 | 2 | <=1 | 2 |

Note: MRSA represent the genotypic confirmation of *S. aureus* carrying the mecA gene. Antibiotic abbreviations used in this study are as follows: FOX, cefoxitin; CHL, chloramphenicol; CIP, ciprofloxacin; CLI, clindamycin; ERY, erythromycin; FUS, fusidic acid (fusidate); GEN, gentamicin; KAN, kanamycin; LZD, linezolid; MUP, mupirocin; HLMUP, high-level mupirocin (256 µg/mL); PEN, penicillin; Q/D, quinupristin/dalfopristin; RIF, rifampicin; STR, streptomycin; SMX, sulfamethoxazole; TET, tetracycline; TIA, tiamulin; TMP, trimethoprim; VAN, vancomycin.

**Supplementary Table S5.** Characteristics of the twenty-two WGS-selected strains.

|  | Sample Type * | Category | Date | Species | Phenotype | Phylogenetic group |
| --- | --- | --- | --- | --- | --- | --- |
| Strain_1 | WW 3 | CLEAN | 20/01/25 | *E. coli* | IND | A |
| Strain_2 | FS 4 | DIRTY | 20/01/25 | *E. coli* | IND | B1 |
| Strain_3 | WW 5 | DIRTY | 03/02/25 | *E. coli* | IND | A/C |
| Strain_4 | WW 9 | DIRTY | 17/02/25 | *E. coli* | IND | A |
| Strain_5 | WW 13 | DIRTY | 03/03/25 | *E. coli* | IND | B1 |
| Strain_6 | FS 19 | DIRTY | 24/03/25 | *E. coli* | IND | B1 |
| Strain_7 | WW 33 | DIRTY | 23/06/25 | *E. coli* | IND | A |
| Strain_8 | FS 7 | DIRTY | 03/02/25 | *E. coli* | ESBL | B1 |
| Strain_9 | FS 16 | DIRTY | 03/03/25 | *E. coli* | ESBL | B1 |
| Strain_10 | FS 16 | DIRTY | 03/03/25 | *E. coli* | ESBL | B1 |
| Strain_11 | WW 25 | DIRTY | 09/06/25 | *E. coli* | OXA | B2 |
| Strain_12 | WW 29 | DIRTY | 16/06/25 | *E. coli* | OXA | E/D |
| Strain_13 | WW 31 | CLEAN | 16/06/25 | *E. coli* | OXA | E/D |
| Strain_14 | WW 34 | DIRTY | 23/06/25 | *E. coli* | OXA | E/D |
| Strain_15 | FS 4 | DIRTY | 20/01/25 | *A. baumannii* |  |  |
| Strain_16 | FS 8 | DIRTY | 03/02/25 | *A. baumannii* |  |  |
| Strain_17 | WW 21 | DIRTY | 05/05/25 | *A. baumannii* |  |  |
| Strain_18 | WW 22 | DIRTY | 05/05/25 | *A. baumannii* |  |  |
| Strain_19 | WW 22 | DIRTY | 05/05/25 | *A. baumannii* |  |  |
| Strain_20 | WW 4 | CLEAN | 20/01/25 | *K. pneumoniae* |  |  |
| Strain_21 | FS 24 | DIRTY | 05/05/25 | MRSA |  |  |
| Strain_22 | FS 24 | DIRTY | 05/05/25 | MRSA |  |  |

Note: *WW=wastewaters; FS= floor swabs*.*

**Supplementary Table S6.** MLST, ARGs, plasmids and phenotypic resistance of WGS-selected strains.

| Strain | Species | MLST (Allele dg/Allele de novo | ARGs detected with ResFinder | Phenotypic Resistance | Plasmid detected with PlasmidFinder |
| --- | --- | --- | --- | --- | --- |
| Strain_1 | *E. coli* ind | 10/10 | blaTEM-176, aadA1, aph(3')-Ia, qnrS1, dfrA14, dfrA1, tet(A), floR | Ampicillin, Chloramphenicol, Ciprofloxacin, Tetracycline, Trimethoprim | ColpEC648, IncFII, IncX1 |
| Strain_2 | *E. coli* ind | NUOVO/540 | blaTEM-1C, tet(A), floR | Ampicillin, Chloramphenicol, Tetracycline | IncFIA(HI1), IncFIB(K), IncFII, IncX1 |
| Strain_3 | *E. coli* ind | NUOVO/2496 | blaTEM-1B, aph(6)-Id, ant(3'')-Ia, aadA1, aph(3'')-Ib, sul1, sul2, dfrA1, mph(B), mef(C), mph(G), tet(A), catA1 | Ampicillin, Azithromycin, Tetracycline, Trimethoprim | IncFIB(AP001918), IncFII, IncI1-I(Alpha) |
| Strain_4 | *E. coli* ind | NUOVO/6778 | blaTEM-1B, aadA2, ant(3'')-Ia, aadA1, aph(3')-Ia, sul3, dfrA12, tet(A), cmlA1 | Ampicillin, Chloramphenicol, Tetracycline, Trimethoprim | N.D. |
| Strain_5 | *E. coli* ind | 542/542 | blaTEM-176, aph(3')-Ia, qnrS1, dfrA14, tet(A), floR | Ampicillin, Chloramphenicol, Ciprofloxacin, Tetracycline, Trimethoprim | IncX1 |
| Strain_6 | *E. coli* ind | NUOVO/58 | blaTEM-1B, aph(6)-Id, aph(3'')-Ib, sul2, dfrA5, tet(A) | Ampicillin, Tetracycline, Tigecyclin, Trimethoprim | N.D. |
| Strain_7 | *E. coli* ind | 4429/4429 | blaTEM-1B, aadA1, tet(A), floR | Ampicillin, Chloramphenicol, Tetracycline, Trimethoprim | IncR, IncY |
| Strain_8 | *E. coli* ESBL | NUOVO/767 | blaCTX-M-8, blaTEM-1B, aph(6)-Id, aph(3'')-Ib, sul2, tet(A) | Cefepime, Cefotaxime | ColpEC648, IncFIB(AP001918), IncI1-I(Alpha), p0111\ |
| Strain_9 | *E. coli* ESBL | 2628/2628 | blaCTX-M-14 | Cefepime, Cefotaxime, Ceftazidime | N.D. |
| Strain_10 | *E. coli* ESBL | 2628/2628 | blaCTX-M-14 | Cefepime, Cefotaxime, Ceftazidime | N.D. |
| Strain_11 | *E. coli* OXA | 3057/3057 | blaOXA-181, tet(A), floR | Ertapenem, Meropenem, Temocillin | N.D. |
| Strain_12 | *E. coli* OXA | 3057/3057 | blaOXA-181, tet(A), floR | Ertapenem, Meropenem, Temocillin | ColKP3, IncFIB(AP001918), IncFII, IncX3 |
| Strain_13 | *E. coli* OXA | 3057/3057 | blaOXA-181, tet(A), floR | Ertapenem, Temocillin | ColKP3, IncFIB(AP001918), IncFII, IncX3 |
| Strain_14 | *E. coli* OXA | 3057/3057 | blaOXA-181, tet(A), floR | Ertapenem, Temocillin | ColKP3, IncFIB(AP001918), IncFII, IncX3 |
| Strain_15 | *A. baumanni* | 3189/3189 | blaOXA-126, blaADC-25 | Ceftazidime/clavulanic acid | N.D. |
| Strain_16 | *A. baumanni* | NUOVO/NUOVO | blaOXA-208, blaADC-25 | No phenotypic resistance detected | N.D. |
| Strain_17 | *A. baumanni* | 2791/2791 | blaADC-25, blaOXA-317 | Ceftazidime/clavulanic acid | N.D. |
| Strain_18 | *A. baumanni* | 2791/2791 | blaADC-25, blaOXA-317 | Ceftazidime/clavulanic acid | N.D. |
| Strain_19 | *A. baumanni* | 2791/2791 | blaADC-25, blaOXA-317 | Ceftazidime/clavulanic acid | N.D. |
| Strain_20 | *K. pneumoniae* | NUOVO/200 | blaSHV-185, OqxA, OqxB, fosA | No phenotypic resistance detected | N.D. |
| Strain_21 | MRSA | 398/398 | mecA, blaZ, str, dfrG, lnu(B), lsa(E), tet(M), tet(K) | Ceftoxitin, Cl*ind*amycin, Quinopristin/dalfoprist, Streptomycin, Tetracycline, Tiamulin, Trimethoprim | rep7a, repUS43\ |
| Strain_22 | MRSA | 398/398 | mecA, blaZ, str, dfrG, lnu(B), lsa(E), erm(C), tet(K) | Ceftoxitin, Cl*ind*amycin, Erythromicin, Quinopristin/dalfoprist, Streptomycin, Tetracycline, Tiamulin, Trimethoprim | rep7a, repUS43} |

Note: N.D. refers to absence of detected plasmids.

**Supplementary Table S7.** SNP analysis of *E. coli* performed with MINTyper (Center of Genomic Epidemiology).

|  | Strain_1 | Strain_10 | Strain_11 | Strain_12 | Strain_13 | Strain_14 | Strain_2 | Strain_3 | Strain_4 | Strain_5 | Strain_6 | Strain_7 | Strain_8 | Strain_9 |
| --- | --- | --- | --- | --- | --- | --- | --- | --- | --- | --- | --- | --- | --- | --- |
| Strain_1 | 0 |  |  |  |  |  |  |  |  |  |  |  |  |  |
| Strain_10 | 16697 | 0 |  |  |  |  |  |  |  |  |  |  |  |  |
| Strain_11 | 41715 | 45696 | 0 |  |  |  |  |  |  |  |  |  |  |  |
| Strain_12 | 41714 | 45695 | **1** | 0 |  |  |  |  |  |  |  |  |  |  |
| Strain_13 | 41714 | 45695 | **1** | **0** | 0 |  |  |  |  |  |  |  |  |  |
| Strain_14 | 41714 | 45695 | **1** | **0** | **0** | 0 |  |  |  |  |  |  |  |  |
| Strain_2 | 10448 | 17164 | 44244 | 44243 | 44243 | 44243 | 0 |  |  |  |  |  |  |  |
| Strain_3 | 7499 | 17678 | 43743 | 43742 | 43742 | 43742 | 10937 | 0 |  |  |  |  |  |  |
| Strain_4 | 16555 | 9695 | 45565 | 45564 | 45564 | 45564 | 17085 | 17498 | 0 |  |  |  |  |  |
| Strain_5 | 9085 | 17771 | 43864 | 43863 | 43863 | 43863 | 12188 | 9405 | 17678 | 0 |  |  |  |  |
| Strain_6 | 16606 | 8661 | 45308 | 45307 | 45307 | 45307 | 17442 | 17860 | 10203 | 17765 | 0 |  |  |  |
| Strain_7 | 8891 | 17684 | 43774 | 43773 | 43773 | 43773 | 12147 | 9045 | 17548 | 6339 | 17600 | 0 |  |  |
| Strain_8 | 16481 | 8679 | 45443 | 45442 | 45442 | 45442 | 17281 | 17857 | 10220 | 17523 | **1252** | 17630 | 0 |  |
| Strain_9 | 16697 | 0 | 45696 | 45695 | 45695 | 45695 | 17164 | 17678 | 9695 | 17771 | 8661 | 17684 | 8679 | 0 |

**Supplementary Table S8.** SNP analysis of *A. baumannii* performed with MINTyper (Center of Genomic Epidemiology).

|  | Strain_15 | Strain_16 | Strain_17 | Strain_18 | Strain_19 |
| --- | --- | --- | --- | --- | --- |
| Strain_15 | 0 |  |  |  |  |
| Strain_16 | 23764 | 0 |  |  |  |
| Strain_17 | 23672 | 24891 | 0 |  |  |
| Strain_18 | 23672 | 24891 | **0** | 0 |  |
| Strain_19 | 23672 | 24891 | **0** | **0** | 0 |
